# Report on the Progress of Human Anatomy and Physiology, in the Years 1843-4

**Published:** 1845-04

**Authors:** James Paget

**Affiliations:** Lecturer on General and Morbid Anatomy and Physiology, and Warden of the Collegiate Establishment, at St. Bartholomew's Hospital


### REPORT ON THE PROGRESS OF HUMAN ANATOMY AND PHYSIOLOGY, IN THE YEARS 1843-4.

### Part II.

By James Paget,
Lecturer on General and Morbid Anatomy and Physiology, and Warden of the Collegiate Establishment, at St. Bartholomew's Hospital.

### NUTRITION.

*In its chemical relations. Use of gelatine in food.* The Amsterdam commission* for determining the nutritive properties of gelatine, as obtained from bones by steam, and used in large quantities in "economic soup" in Dutch public institutions, have confirmed the conclusion of the commission of the Paris Institute, that it has had hardly any nutritive properties when taken alone; and, in regard to the important point left unsettled by the French commission, namely, whether, when added to other kinds of food, gelatine contributes to the total amount of nutriment, they have also come to a negative conclusion. None of the three dogs to whom considerable quantities of it were, for several long periods, given both alone and in connexion with bread aud potato-parings, was found to have derived the least benefit, or to have gained any increase of weight from it.

[The experiments were accurately enough made, and warrant the conclusions, as far as "economic soup" and dogs are concerned. But they are so opposed to the results of common observation of the nutritive value of jellies and other like gelatinous food, that some fallacy must be suspected in both these and those of the French commission. Either dogs are improper subjects for such experiments, or, more probably, the mode of preparation decomposes the gelatine. The action of the hot steam to which the bones, already boiled, are subjected for about seven hours in each of several days, may effect a part of the change by which, when it is complete, gelatine loses its power of *setting*, and in which the arrangement of its elements may be so altered, that they cannot be reconstructed in a nutritive form.]

*Transformations of the sugar in food.* M. Chossat† found that of many birds fed on sugar alone, none lived more than sixteen days; and he thought he observed that in those which had copious bilious evacuations, no unusual quantity of fat was accumulated in the body; but in those in which these discharges did not occur, fat was abundantly formed. He assumed, therefore, that the sugar is, under varying circumstances, sometimes converted into the constituents of fat, and sometimes into those of bile. But the experiments of M. Letellier,‡ which were more carefully made, contradict these. Their results were, that among seven turtle-doves fed on cane-sugar and bread with water, (coagulated albumen having been added,

* Het Instituut, No. 2, 1843, pp. 97-114; imperfectly reported in the account of the sitting of the Académie des Sciences, 11 Mars; in the Gazette Médicale, 16 Mars 1844.

† Gazette Médicale, 21 Oct. 1843, from the Acad. des Sciences, Seance du 16 Oct.

‡ Annales de Chimie et de Physique, Juin; and Ann. des Sciences Nat., Juillet, 1844.

in two cases, after the sixth day,) not one possessed, at the time of death, the average quantity of fat: their general average of fat was nearly 60 per cent. less than that found in healthy individuals, i. e. the average in health was found to be 15·8 per cent., and in those fed on sugar only 6·3. Yet the fæcal evacuations had been, in most cases, moderate. But the sugar, though it did not increase the fat, served towards maintaining the temperature of the body and the average production of carbonic acid. The quantity produced by these birds, on ordinary diet, was 13.2 grains per hour; during seven days' starvation, it was 6·65 grains per hour; and during three days' diet of sugar, 11·08 grains. In turtle-doves fed for six days on butter, the quantity of fat found after death was scarcely more than in those who had died on the diet of sugar without albumen; and the quantity of carbonic acid produced by them was 9·08 grains per hour. [Still these experiments of M. Letellier, though they may prove that sugar alone will not keep pigeons alive nor increase their fat, do not disprove that, under favorable circumstances, fat is formed from sugar. Such a transformation is proved, by the experiments of Huber and Milne Edwards on bees, which formed wax while feeding on pure honey; and to their evidence may now be added that of Grundlach.* He fed bees on sugar-candy in water, and they formed wax; from twenty pounds of honey, also, they formed a pound of wax.]

In a controversial note on this and other subjects,† Liebig quotes a letter from M. Demesmay, who states that the result of abstracting from the food of fifty-eight cows a certain quantity of oil-cake, which contains from 10 to 15 per cent. of oil, and substituting for it an equal weight of beetroot-molasses, was a more rapid fattening and a more copious production of milk. And to these evidences of the transformation of saccharine into fatty substances, it may be added that butyric acid may be formed in the fermentation of sugar,† and that M. Avequin,‡ has noticed that the quantity of that crystalline wax which forms on the exterior of the sugar-cane, (and which he has named Cerosia,) always bears an inverse proportion to the quantity of sugar within the cane.

*Production of fat in the animal body.* Besides the experiments above mentioned, others have been performed by M. Persoz and M. Boussingault. Those of the former,§ on the fattening of nine geese with maize, appear to prove that sometimes the weight of fat formed during the fattening exceeds the whole increase of weight of the body. He supposes, therefore, that the geese not only assimilate all the oily matters of the maize, and transform some of its starch and saccharine matter, but also transform a certain portion of their own tissues into fat. During the fattening, the blood becomes highly charged with oily matter, and much of its albumen disappears or is transformed. The experiments of M. Boussingault‖ afford evidence that some nutritive substance analogous to fat must be contained in the food in order that the fatty substances of the secretions and tissues may be duly formed.

*Relation between food and excretions.* M. Boussingault¶ considers that he has proved that in three days a horse and a cow each discharged in excrement and urine (and the latter in milk also), from 356·5 to 418·5 grains less of nitrogen than they had taken in their food. This quantity, therefore, he supposes must have passed off in respiration and transpiration in the free state. [But it is only presumed that the weight of the animals remained the same; and both the food and

* Geschichte der Beinen, 1842.

† Annalen der Chemie und Pharmacie, Oct. 1843.

† See last Report, p. 15.

‡ In Mulder's Physiol. Scheikunde, p. 271.

§ Report of the Académie des Sciences, 12 Fevrier, 1844; in the Gazette Médicale, 17 Fevr. I suspect that in that fatty degeneration of the muscles which takes place when they are not exercised, the change is not a removal of the muscular fibre and deposition of fat in its place, but a transformation of the fibrine, of which one of the products is fat; that it is, therefore, a chemical not a nutritive process by which the change is effected. The nature of such a change is illustrated by Wurtz's observation (p. 250); and its occurrence is made very probable by the linear arrangement of the particles of fat, in the places of the former muscular fibres, on a plan different from that existing in any other form of fat.

‖ Annales de Chimie et de Physique, Oct. 1844.

¶ Annales des Sciences Naturelles, Avril, 1844. Extract from M. Boussingault's work 'Economie rurale dans ses rapports avec la Chimie, &c.'

excretions were analysed by samples, so that small errors would be greatly multiplied in the general result; and no account is taken of what may have been discharged from the skin in hair, epidermis, &c.—an omission which must in many similar cases have left errors unaccounted for.]

A case of voluntary total abstinence from food and drink for ten days, is related by Dr. Casper.* The patient was thirty-six years old. For the first five days he suffered little, and confessed neither hunger nor thirst; during this time also he passed no fæces and very little urine. After this he became thinner and paler, his sight was weak, he had occasionally ringing in the ears, his speech was indistinct, his breath smelt unpleasantly, he discharged only a little urine, his abdomen sank in, and he was very weak. These conditions were increased on the ninth day, and he could not resist taking a little sugared water. In the night of the tenth day hunger, which he had before hardly felt, returned irresistibly, and he took food, and recovered. Mitscherlich examined the urine which was passed at the middle of his abstinence, and found that it did not differ from that of a healthy person.

*Nutrition in its relations to structure. Development of cells.* In the last Report,† the observations of Mr. Macleod are quoted, from which it appeared that in the chick, the blood-corpuscles are not developed by the formation of a cell round a preformed nucleus, but that each corpuscle is produced by the enlargement of a single granule, from the contents of which the nucleus is subsequently formed. Some other observations—by Schwann, on the development of the chorda dorsalis, by Vogt, on the development of new cells in the intercellular substance of the cartilage, and by others — had also afforded examples of cells formed before those cells within them which are regarded as their nuclei; and now, in an elaborate essay on the development of cells in general, Dr. Karsten‡ maintains that this is the regular plan of cell-development; that, at the first, each cell is a punctiform vesicle, which, subsisting on the surrounding substance, grows into a simple cell, and forms within itself some peculiar substance, either a secretion, or the rudiments of a new cell (or nucleus), or a new organism, as an ovum or gemmule.

A large portion of the evidence for this view is drawn from observations on vegetable cells. In that portion which relates to animal physiology, the chief examples are drawn from the development of the ova, gland-cells, the cells of the chorda dorsalis, cartilage, epidermis, pigment, and feathers.

In the *development of the ovum*, R. Wagner believed, from observations on the ovaries of the Agrio Virgo, that the germinal spot is the part of the ovum which is first formed, and that the germinal vesicle, vitellus and vitellary membrane are formed in succession around it; and it was in great measure through these observations that Schwann was led to adopt for the development of animal tissues the theory which Schleiden was supposed to have demonstrated for those of vegetables. Karsten considers that Wagner was deceived by the fineness of the membranes of the ovaries of the Agrio, which prevented his seeing that each of the follicles consists of two membranous cylinders—an internal one inclosing the ova, and an external one surrounding it; and that these have between them cells, which, through the delicacy of the membranes, appear as if they were within the internal cylinder, and were ova, but which disappear when the real ova are formed. The error cannot be committed with the ovaries of Lepidoptera: and among the best for examination are those of the Bombyx Mori, in the chrysalis state. The blind end of the internal cylinder may be found full of minute *simple vesicles;* and some of these may be traced, as they are examined further from the end, enlarged and filled by a granular blastema, and having in them a new cell which lies in the blastema. This is the germinal vesicle imbedded in the vitellus, and having the originally simple cell for the vitelline membrane. The same grades of development may also be traced in the ova of mollusca, if they are examined before they are filled with yelk.

The *development of secernent gland-cells* affords another example of the same

* Casper's Wochenschrift, June 8, 1844. † Page 5. ‡ De Cellâ Vitali; Berlin, 1843.

plan of development. Some of Mr. Goodsir's observations agree with this view; and Karsten says the best evidence of it may be found in the follicles of the liver of mollusca and crustacea. In these may be seen, between the tunica propria and the central canal of the follicle, a great quantity of cells, surrounded by a liquid blastema, and containing a variety of granules, vesicles, and lesser cells; and there may he traced a series of formations, from the minute granule to the perfect cell, which may contain one or more cells, such as would be called nuclei, with one or more minute vesicles or nucleoli, in them. But in no member of the series will there be found a preformed cytoblast or collection of granules.

Schwann's and Vogt's observations on the cells of the chorda dorsalis and cartilage appear to Karsten (who has confirmed them) to support the existence of the same plan of development in those tissues; and he shortly describes the same as obtaining in the epidermis, pigment, and the rudiments of the feathers.

He denies also that, in either vegetable or animal growths, cells are ever multiplied by either generation or partition. He says of the apparent germination of the sporules of the Saccharomyces Cerevisiæ, and others of the lowest algæ (in which alone this process has been supposed to occur), that the process is not one of mere sprouting out of a part of the wall of the previous cell;* but that it consists in the development of a granule, which at first lies obscurely between the two membranes of which the cell is composed. As this granule is developed into a vesicle or cell, it expands over itself a part of the outer membrane of the previous or parent cell, till there is produced the appearance of two double-membraned cells united by a narrow constriction of their common outer membrane but having their own cavities distinct. And as this goes on, the secondary cell has various contents formed within it; and may, by a repetition of the same process, develope a tertiary cell in its wall. The process may be best traced in the growing sporules of Phragmotrichum; but it may also be examined in the Saccharomyces, in which an external membrane, enveloping each apparently simple cell, may be demonstrated by moistening it after it has been dried for some time.

*Anatomy of cells.* Numerous examples of molecular movements of the granules in cells are described by Mr. Addison.† They are seen especially in the pale corpuscles of the blood, pus-globules, and mucus-globules, which all appear full of minute molecules in energetic movement. Prof. Rathke,‡ also, has often observed similar movements of particles within the nuclei of the cells of the ova of the frog, river crayfish, &c. But he shows that these movements depend, in all probability, on the currents produced in the fluid contents of the nuclei, by the imbibition of some of the water in which they are examined; for in every case in which they happen, the nuclei and cells become gradually larger; and, by examining them in oil, they neither enlarge nor exhibit the molecular movement. In this way may be probably explained the molecular movement of the pigment-granules of the choroid as seen within the cells. But the facts will not explain the observation of Dr. Sharpey,§ who has seen pigment-granules coursing round and round within the spherical epithelium-cells of a tadpole, and making the complete circuit of its cavity.||

### ORGANS AND FUNCTIONS OF EXCRETION.

*Skin and its appendages.* A complete examination of the structure and some of the functions of the skin and its appendages, with many original observations, has been published by Professor Krause.¶ Of course, the sizes and weights of everything that can be so estimated are given. In explanation of the colour produced in the epidermis by nitrate of silver, and supposed to depend on the de-

* See also Sharpey, in Quain's Anatomy, p. lii.

† Provincial Med. and Surg. Journal, March 9 and June 5, 1844.

‡ Müller's Archiv, 1843, Heft vi.

§ Quain's Anatomy, 1843, p. lvi.

|| On all that relates to the healing processes and inflammation, I refer to the excellent Reports on the subject by Mr. T. Wharton Jones, in the last April and July Numbers of this Review. For many experiments on the healing of fractures, see Lebert, "De la formation du Cal," in the Ann. de la Chirurgie, Fevr. 1844.

¶ In the unfinished article *Haut* in Wagner's Handwörterbuch der Physiologie, Lief. vii.

composition of the tissue, Krause says that if thin-cut layers of epidermis soaked in a solution of nitrate of silver be exposed to the light, and then made transparent by acetic acid, their texture may be seen to be unaltered, but there are very dark granules from 1-1000th to 1-1500th of a line in diameter, on the outside of the larger cells, which are, no doubt, chloride of silver and reduced silver, and to these, not to a decomposed tissue, the change of colour is due.

Krause says also, that the colour of the cuticle of the Negro does not depend (as Henle supposes,) on pigment-cells, like those of the pigmentum nigrum, lying between the cutis and rete, and mingled with the cells of the latter, but, chiefly, on the colour of the proper nuclei and cells of the epidermis. There are indeed some few pigment-cells mingled with the proper cells of the middle and superficial layers of the epidermis; but they are distinguished from those of the pigmentum nigrum by containing far fewer pigment-granules, and by having always a dark (not a clear) nucleus. The colour depends especially on the dark- or almost black-brown colour of the nuclei, whether free in the deep layers of epidermis, or surrounded by cells. They have dark nucleoli, sharp outlines, appear only very obscurely granular, and cannot be broken into smaller pigment-granules. The cells surrounding them may be seen: in the deeper layers, they also are uniformly dark, though less dark than the nuclei. In the middle and superficial layers, the nuclei, as long as they can be seen, are still dark; the cells are much paler, but brownish and darker than in the corresponding layers in uncoloured persons.

The so-called *Tyson's glands*, the little white elevations which are usually found round the corona glandis of the human penis, and which, after many disputes, have been usually considered as the secernent follicles of the *smegma preputii*, have been carefully examined by Dr. G. Simon.* They are, he says, no more than small round elevations of cutis covered by papillæ and epithelium. They consist of fibro-cellular tissue like that of the rest of the cutis; and the papillæ on them have no peculiar characters. The only function that can be ascribed to them is that of increasing the sensibility of the glans. The only organs which Simon could find for the special secretion of the *smegma*, (and these are not constant,) are whitish corpuscles lying in or beneath the cutis, which, with the microscope, appear as small roundish sacculi, closed below, and opening by a narrow orifice on the surface, and containing a white substance. These are usually situated on or behind the corona glandis, in front of or near the frænum, and sometimes on the anterior surface of the glans itself. Two or three may be found, or, in a few cases, as many as six.

*Cutaneous perspiration.* Krause† has managed to collect, with great care to avoid mixture, a small quantity of pure cutaneous perspiration from the palm, in which, as is well known, there are no sebaceous glands. The fluid collected, yielded with boiling ether some small globules of oil and crystals of margarine: it was acid, but after twenty-four hours became alkaline by the development of ammonia. In another experiment, he found that the tissue of the epidermis, independent of the fatty matter secreted on its surface, contains a fatty substance.

He has also endeavoured to number and measure the sweat-glands. As an average, he says, it may be estimated that in each superficial square inch of the body there are 1000 orifices of glands of 1-6th of a line in diameter; the greatest and least numbers in this space being in the palm 2736, in the sole 2685, in the cheek 548, in the neck, back, and nates 417. The whole number, therefore, excluding the axillæ, in which they are peculiarly large and thick-set, may be estimated at about 2,381,248. Accepting these numbers, and supposing each gland to be occupied by a column of fluid presenting at the orifice a hemispherical surface, 1-56th of a line in diameter, (the size which Krause found by measurement in some drops in a warm and moist but not *sweating* skin,) then the whole of the glands would present an evaporating surface of 7·896 square inches. Hence it is probable, (according to ascertained laws of evaporation, and from experiments purposely made,) that a portion only of the fluid discharged by cutaneous exhalation is produced by these glands; for there could not be more than 3365 grains evaporated

* Müller's Archiv, 1844, Heft i. † L. c. p. 146.

in the twenty-four hours from such a surface, under favorable circumstances; whereas Seguin's experiments show that the daily cutaneous transpiration varies from 5·93 to 10·465 grains per minute: probably, therefore, much of what is discharged by the skin, passes by simple transudation through the epidermis, and evaporation from its free surface.

Experiments by Valentin,* made with great care on his own person, afford evidence concerning the total daily amount of transpiration, both cutaneous and pulmonary. Taking three days of ordinary life in September, weighing himself while naked, fifteen times a day, and weighing all his ingesta and sensible excretions,—the averages of the three days gave,—nutritive matter taken, 45325·5 grains; excrement, 2956·3 grs.; urine. 22439·3 grs.; perspiration, 19327·4 grs.; i.e. the ingesta being taken as 1, the excrement was ·065, the urine ·503, the perspiration ·422. But there were differences in the days; in the first, the relation between the ingesta and the excretions was as 1·097 to 1; in the second, as 1·028 to 1; on the third, as 1 : 1·090.

The hourly amount of transpiration was at some times 4½ times as great as at others; the greatest difference being caused by whatever excited sweating or a perceptible moisture of the skin, e.g., on the same day, the hourly amount was, after taking two cups of coffee, and during gentle perspiration, 1213·65 grains; in the forenoon, in pretty active exercise and sweating, 1402·75 grains; and in the evening, during copious sweating from exercise, 2056·85 grains; but, while writing quietly in the forenoon of the same day, it fell to 858·7 grains; and three or four hours after dinner, was only 509·95 grains. Of all things, none influenced the transpiration so much as rest and bodily exercise; even when the latter did not produce evident sweating, its effect was considerable. After eating also, transpiration was generally increased; and its minimum was observed during fasting and rest in a cool temperature. During the night and in sleep, the transpiration was diminished, but not more than in rest during the day. Mental exercise had no obvious influence.

Experiments by M. Magendie† confirm those by M. Fourcault and others, on the effects of covering the skins of animals with varnishes impermeable to air. The animals always died as if asphyxiated, with their hearts and lungs gorged with blood; and during life the temperature of their bodies fell gradually 18°, 24°, and as much as 36° below the ordinary standard. The same effects were produced by inclosing animals, (all but their heads,) in dresses of caoutchouc cloth.

*Structure of the nails.* Reichert‡ considers, as Kohlrausch§ also does, that the streaks described by Henle in sections of nails, as indicative of their laminated structure, are only cracks and seams produced by the knife; and says that their direction is always determined by that in which the section is made. To him the nail appears homogeneous; except for those dark spots which Kohlrausch and Krause suppose to be remains of nuclei, but which Reichert considers to be vacant spaces in the nails.

Reichert further considers, that the only matrix of the nail is that part of the cutis which forms the posterior half of the upper wall of the proximal semilunar groove, and the angle and lower wall of the groove, and that which lies beneath the lunula. There is a layer of cells similar in kind, but in different stages of development on the whole of the surface of the cutis above and below the nail; but the destination of the cells on different parts of this surface is very different, and it is only on the part indicated above that they are developed into nail-substance. Above the nail, they are developed into the thin layer of epidermis, which forms a fringe upon the borders of the nail. On all the surface of the cutis anterior to the lunula, (on what is called the *bed of the nail,*) the cells form a layer like epidermis, (i.e. according to Krause, like the deepest layer of the epidermis in other

* Physiologie, Bd. i, p. 7161. The details are given in Valentin's Repertorium, Bd. ix, and some in the Medical Gazette, July 19, 1844.

† Constantin James, 'Voyage à Naples avec M. Magendie;' Gazette Médicale, Dec. 6, 1843.

‡ Müller's Archiv, 1843; Jahresbericht, pp. 270-9.

§ Göttingische Gelehrte Anzeigen, Ht. xxiv, p. 229.

parts of the body,) between which and the under surface of the substance of the nail, there is a distinct line of boundary, the long diameter of those of its cells which are immediately adjacent to the nail being *perpendicular* to its axis. This arrangement exists as far forwards as the nail is closely adherent, i.e., to within a line of the part at which the nail and cutis separate ; for this line's breadth, a thin layer of ordinary epidermis is prolonged backwards under the nail, as another layer is prolonged forwards on the inferior surface of its free part.

The cells formed on the surface of the proper matrix of the nail are, in their early conditions like those of epidermis; but as they go on to form nail, they become larger, more transparent, oval and flat; their nuclei (which according to Krause are very evident, by their darkness, in the negro,) become smaller and at last disappear, and gradually the traces of their own outlines are lost, as they unite into the compact, uniform, and nearly transparent, proper substance of the nail. It is only the cells formed at the angle of the groove which, lying parallel to the plane of the nail, have from the first a right direction forwards; those formed on the upper and lower walls, only gradually assume the same direction. Hence the surfaces of vertical sections of the root of the nail are slightly marked in a penniform manner. The whole nail therefore may be described as formed from the cells produced at the matrix ; and as sets of these are successively produced and coalesce, the older ones are pushed forwards over the layer of epidermis-like cells which covers the whole surface of the bed of the nail.

*Structure of hair.* Krause's* description of the structure of the nails, tends to show their exact analogy with epidermis; and he thinks the hairs may also be regarded as epidermoid tissues, their cortical portion being analogous to the outer or horny layer, their medullary portion to the inner layers of epidermis, and their shape being due only to that of their matrix. The root-sheath of the hair, he says, is only the epidermis of the follicle; its outer layer is the continuation of the deep and middle layers of epidermis, and consists of nuclei and roundish or polygonal cells, which are especially distinct in the negro, and are set vertically to the wall of the follicle; the inner layer, (or inner root-sheath,) is continuous with the outer horny layer of epidermis, and is composed of long flat cells with few or no visible nuclei. It is very apt to tear in long fibres; the holes in it, which led Henle to call it a fenestrated membrane, are produced by the manipulation.†

### KIDNEYS AND THEIR SECRETION.

*Composition of the urine.* In a highly interesting paper on the constitution of the urine Liebig‡ maintains the following points:

A. That neither lactic acid nor any lactate exists in healthy urine; the evidence being, 1, that hitherto no example is known of lactic acid being produced by the decomposition of a nitrogenous substance; 2, that the urine of the herbivora, in which lactic acid or its salts might be expected, (if they existed in that of the carnivora,) does not contain either; 3, that lactic acid has never yet been clearly detected in the urine of men or carnivora ; 4, that the carnivora take no food from which lactic acid could by transformation, be produced; 5, that fresh urine will not dissolve the smallest quantity of barytes, though lactate of barytes is easily soluble in water ; 6, that in various and the most careful experiments, it has been impossible to detect even a trace of lactic acid in large quantities of putrid urine, in which, if it had existed when fresh, it could not have been altered by putrefaction, and if it had not existed when fresh, it might perhaps have been produced by putrefaction. An organic acid was produced in putrefaction, but it was acetic acid combined with a resinous highly azotised substance.

B. Hippuric acid is a constant constituent of healthy human urine ; for 1, benzoic acid is obtained (as Proust observed,) with acetic acid, by distilling urine

* L. c. p. 125, &c.

† On the chemical composition of the hair, I can only refer to the elaborate analysis of J. F. J. van Laer, in the Scheikundige Onderzoekingen ; Utrecht, 1842. Analyses of it are given in Schmidt's Jahrbucher, Oct. 1843; the Medical Times, Feb. 3, 1844; and, briefly, in Mulder's Phys. Scheikunde.

‡ Ann. der Chemie und Pharm., Mai; and Lancet, June 1-8, 1844.

with sulphuric or hydrochloric acid; but, 2, this benzoic acid cannot exist as such in the fresh urine; for benzoic acid is converted in the organism into hippuric acid; and the hippuric acid known to exist in the urine of herbivora yields benzoic acid when it is decomposed; and 3, the existence of hippuric acid may be clearly proved in even small quantities of fresh urine, by evaporating it to the consistence of syrup, mixing with it some hydrochloric acid, and agitating it with ether, which dissolves the hippuric acid.*

The hippuric acid thus obtained, cannot be derived from the decomposition of benzoic acid taken in the food, (for probably none of man's food contains any;) it is formed in the body from the non-nitrogenized aliments. The acetic acid does not exist in fresh urine; but it and the resinous substance with which it is combined may be regarded as the products of the decomposition of the colouring matter of the urine.

C. The acid reaction of healthy urine is due to the presence of the acid phosphate of soda, and the mode in which this salt is produced is as follows: alkaline phosphates are taken in meat, flour and grains; none of these contains any free alkali; and it is from these phosphates, and not from any free alkali or alkaline carbonate, that the chyle, lymph, and blood, derive their alkaline reaction. Now, among the remarkable properties of the bibasic phosphates of soda and potass are their relations to uric and hippuric acids. Both these acids dissolve very easily in water, to which common phosphate of soda has been added, and with their solution, the phosphate loses its alkaline, and assumes an acid, reaction. And thus, when the uric and hippuric acids are formed in the organism, they combine with the soda of the alkaline phosphate, forming the highly soluble urate and hippurate of soda, and an acid phosphate of soda.

D. But besides this, there is another cause by which the acidity of the urine is maintained and increased. The urine of man and the carnivora contains a large quantity of sulphates; but their food does not contain either those salts ready-formed, or any oxygen-compound of sulphur. The sulphur which it does contain, or (which comes to the same thing,) the sulphur of the transformed tissues, must therefore combine with oxygen in the body, and the sulphuric acid thus formed, combining with part of the alkali of the alkaline phosphates, converts them into acid phosphates, and thus maintains and increases the acidity of the urine.

E. It follows that whether the urine will be acid or not, depends upon the nature and quantity of the *bases* taken with the food. If the amount be sufficiently large to neutralize the uric, hippuric, and sulphuric acids formed by the organism, and the acids supplied by the food, the urine must be neutral; if the amount be more than enough, the urine must be alkaline; if less, acid. And hence, no physiological or pathological inference can be drawn from our examination of the urine, unless an account be taken of the inorganic acids, salts, and bases taken with the food.

An exception to the rule that carnivora alone produce uric acid exists in the case of butterflies, (and other lepidoptera?). Heller has discovered that in proportion to the weight of their bodies they of all animals produce the greatest quantity of uric acid. Their urine is analogous to that of serpents and predatory birds, containing as a chief constituent urate of ammonia; it is principally a product of the metamorphoses which go on in the pupa state, and the red or yellow fluid which they discharge soon after being hatched is chiefly urate of ammonia. The secretion continues in after life.†

### VASCULAR GLANDS.‡

*Spleen.* Dr. Julian Evans,§ together with many confirmations of the descrip-

* Liebig estimates the quantity of benzoic acid in the urine to be equal to that of the uric acid. Dr. Golding Bird has never found it exceed one third of the quantity of the latter. (Med. Gazette, Aug. 23, 1844.)

† Oesterreichische Med. Wochenschrift, Sept. 14, 1844.

‡ Among the notices of these mysterious organs recently published, is an account of the microscopic structure of them all, by Dr. Oesterlin, in his Beiträge zur Physiologie, Jena 1843, 8vo. The results he has arrived at are not, however, so definite or important but that the further account of them may be deferred till, in the next Report, an account is given of the work of Mr. Simon on the Thymus and Thyroid Glands.

§ Lancet, April 6, 1844.

tions of Malpighi, Müller, and others, has given a more complete account than yet existed of the cells of the human spleen. They are, in comparison with its parenchyma, smaller and less capable of distinction than in the graminivora; probably from one-third to half a line in diameter, and pentagonal or hexagonal in form. They are frequently found filled with coagulated blood; and, in this condition they give the spleen a granular appearance. But the chief peculiarity of his account is, that he describes [what certainly needs confirmation, for they must be very difficult to demonstrate] a set of transparent vessels, of less diameter than the small splenic corpuscles, (i.e. less than 1-7000th of an inch,) and apparently arising from them: he believes these to be lymphatic vessels, and that they gradually unite and form trunks which can be traced from the parenchyma into the Malpighian bodies, (which he considers to be lymphatic glands,) from which, after numerous convolutions, they emerge fewer in number but larger, and pass through the pedicle by which the body is attached.

A case is recorded* in which a man lived in good health for thirteen years after removal of the spleen.

Schwager-Bardeleben,† from his experiments on the extirpation of the spleens of animals, has obtained (as others before him have,) scarcely more than negative results. Those who survive the operation appear quite unaffected by it; so also it is with those from which the thyroid body has been removed, (except that in one rabbit the venereal appetite seemed to be increased.) Even when both have been removed, no evident effect is produced upon any of the functions of the animals that survive; but indeed few survive both operations. The removal of the spleen does not produce impotence; and the author has never seen the spleen reproduced. But, according to the continued investigations of Mayer,‡ some slight changes are produced. He says that after the spleen has been extirpated it is usual to find all the mesenteric glands more or less swollen and blood-red, blue, or blackish. Moreover, he has often found the small lymphatic glands, near the part where the splenic artery has been tied, marked with the bloody spots, in which at last capillary networks form; and he believes that then several such glands unite and form the new spleen.

### SKELETON.

M. Breschet§ says that he has seen ten cases in which the human malar bone was composed of two pieces, a superior or orbital, and an inferior, jugal, or zygomatic portion; presenting an analogy with the normal formation in some of the quadrumana and in several other mammalia, and making it probable that the bone is developed from two osseous nuclei.

Dr. Wilbrand|| describes and figures a case in which a *supra-condyloid process,* like that described by Dr. Knox,¶ but less perfect, was found on the human humerus. It arose from the front and inner aspect of the humerus, a short distance above the inner condyle, and, like a rose-thorn in shape, about six lines long and two lines in diameter it extended towards the inner condyle, to which its extremity was connected by a fibrous band; the brachial artery and median nerve after running past the inner side of the process went under the ligament to their ordinary position at the bend of their elbow.

He describes also a process on a femur, which he calls the supra-condyloid process of the femur, and considers as the analogue of the process found in certain edentata, rodentia, pachydermata, and others. In these there is a process of considerable size on the outer aspect of the thigh, at the middle, or a little above or below the middle of the shaft. The subject in whom he found a rudiment of a similar process was a strong man. The process was

* Gazette Médicale, No. 28, 1844; and Oesterr. Wochenschrift, Sept. 21, 1844.

† Gazette Médicale, 23 Mars, 1844. Report from the Académie des Sciences; Seance de 18 Mars.

‡ Med. Correspondenz-Blatt Rhein. und Westphal. Aerzte, 1843, No. 5; and Schmidt's Jahrbucher, Januar. 15, 1844.

§ Annales des Sciences Naturelles, Janvier, 1844.

|| Ueber Processus supracondyloideus Humeri et Femoris; Giessen, 1843, 4to.

¶ Edinburgh Medical and Surgical Journal, 1841, vol. lvi; and Medical Gazette, July 7, 1843. See also, on similar subjects, his numerous and interesting "Contributions to Anatomy and Physiology" in the Med. Gaz., Nov. 4, &c. 1843.

situated at the attachment of the short portion of the biceps femoris. It was one and a half inches long, four lines thick, and projected outwards nearly three quarters of an inch. It was covered by the persistence of the femur, and a rather large nutritive artery of the femur passed through it; it had not at all the aspect of a morbid exostosis.

### VOICE.

MM. Pétrequin and Diday,* after, as they believe, satisfactorily disproving all previous explanations of the falsetto voice, maintain that in it "the glottis places itself in such a state that the vocal cords can no longer vibrate like reeds. Its contour represents the embouchure of a flute, and it is not by the vibrations of the aperture, but by those of the air, that the sound is produced." They do not explain how the assumed rigidity of the lips of the glottis by which these vibrations are prevented is produced; [it would be very difficult to do so:] but they maintain their theory by the following statements: 1. There is an analogy between the tones of the falsetto voice and those of the flute, from which the former are often called *fluty*. 2. There are but two modes in which voice can be produced: by the vibration of the vocal cords and by that of the air; and since the chest-notes are produced by the former, the falsetto must be produced by the latter. 3. Bass voices have commonly no falsetto notes [?]; because the aperture of the glottis is too large for the air to be thrown into vibrations in passing through it. 4. High chest-notes easily pass into the corresponding falsetto notes when we try to soften them; for when we wish to diminish the loudness of any note we are singing, the glottis is instinctively constricted to prevent the note from falling in consequence of the diminished force of the current of air; but when the note which is being sung is high, and the ligaments already very tense, a reduced current of air could not make them vibrate if they were still more tense; the air therefore, instead of making them vibrate, vibrates itself as it passes between them, and the glottis is changed from a reed-like to a flute-like instrument. 5. In the same manner when we try to strengthen a low falsetto note, it unavoidably assumes the character of a chest note, by the lips of the glottis passing from the rigid state to that of vibration,—from the state in which the air alone vibrates in passing between them, to that in which themselves vibrate. 6. The difficulty of passing imperceptibly in an ascending or descending scale, to or from the falsetto notes, indicates that the state of the glottis in the two kinds of voice is wholly different. 7. The supposed change, from the vibrating to the rigid state of the lips of the glottis, may be imitated and illustrated with a reed-instrument, such as a bassoon. Its ordinary notes are like chest notes—but if, while sounding them, the reed be suddenly taken hold of and held with forceps so as to prevent its vibrations (though nothing else be altered,) the notes become acute, soft, and whistling—they pass from reed-notes to flute notes—from those like the chest-notes to those like the falsetto.

### NERVOUS SYSTEM.

*General anatomy. Structure of the nerve-fibres.* Reichert† confirms Volkmann and Bidder's account of the speciality of the sympathetic nerve-fibres, their distinctness in size and structure from the cerebro-spinal.‡ He believes also that the tissue which invests the smallest fasciculi of the sympathetic fibres of the higher vertebrata, (that which has been variously described as filamentous epithelium, fibro-cellular tissue, *formatio granulosa*, &c.,) is a transparent, finely granulated membrane, which has a peculiar tendency to wrinkle, and sometimes to separate, in a longitudinal direction, so as to assume the appearance of a fibrous texture.§

* Gazette Médicale, Fevr. 23, and Mars 2, 1844.

† Müller's Archiv, 1844; Jahresbericht, p. ccvi.

‡ See Report, 1842, p. 34. But Valentin still maintains the absence of any distinction between the two sets of fibres, in Müller's Archiv, 1844, Heft iv, p. 395.

§ In like manner he describes the tissue connecting the vessels and other elementary parts of the kidney, as Mr. Bowman does who calls it the matrix, as a uniform structureless substance which has even less tendency than the similar connecting tissue in most parts of the body has to wrinkle itself or break up so uniformly as to appear like fibro-cellular tissue. In all this his view coincides with that of Dr. Todd and Mr. Bowman. (See last Report, p. 5.)

*Central ends of nerve-fibres.* In a monster without either brain, medulla oblongata, or spinal cord, Dr. Lonsdale* examined the central extremities of the roots of several of the cerebral nerves, (from the 5th downwards,) which were hanging free and unattached in the cranial cavity. The extremities were enveloped in a delicate membrane of filamentous tissue, but after removing this and numerous granules which surrounded the nerve-fibres, it was quite evident that the latter, in every instance in which they could be examined, formed loops. Each fibre, after passing to the central end of the fasciculus, then turned back upon itself, and could be traced down the fasciculus towards the periphery. The fact has peculiar interest in that it adds probability to the opinion of Valentin, Carus, and Klencke,† that in the normal state the nerves in their cerebral central extremities (as they are called,) form loops, analogous to those formed at their peripheral extremities.

But that this is not the only mode in which the nerve-fibres are related to the centres would appear from the observations of Dr. Will‡ on the nervous ganglia, and the origins of the nerves in invertebrata. He states that the primitive nervous fibres terminate or commence in certain nerve-corpuscles or ganglion-corpuscles within the cephalic and other ganglia. The general results of his examinations|| (so far as they are likely to be soon applicable in human physiology,) are, that the nerves enter and leave the ganglia through constricted apertures or meshes in its external investment, the fibres of which are continuous with those of their neurilemma, and with others by which the ganglia are partitioned. The ganglia contains besides the nervous fibres and the nerve- (or ganglion-) corpuscles, a granular substance filling up the spaces between them, and often coloured by pigment, and various cells. There are two kinds of ganglion-corpuscles. In the one kind, the space between the investing membrane (the secondary-cell of Henle,) and the cell is filled by a pellucid hyaline substance, which coagulates by the action of water or acids. Each of these corpuscles has always one appendage, a simple tube, which never divides into branches, but may be traced into direct continuity with a primitive nerve-fibre, or tube. In the ganglion-corpuscles of the second kind, there are numerous minute cells without nuclei in the clear substance within the investing membrane; and they have generally several appendages, which neither are nor contain tubes, but are striated, and consist throughout of fine fibres. In their course they divide into two or three branches, which may again branch, and even break up into their component fibres. The larger branches have, at various distances, varicose enlargements; and the smallest ones run together into very small ganglion-like swellings, with dark central spots like nuclei, from which again similar fibres proceed in all directions.

When the nerves enter at the cephalic margin of the ganglion, after passing through the constricted aperture, they spread out a little, and enlarge, and finely granular substance lies between them; when they leave it they become closer again, and similar granular matter accompanies them for some distance in their sheath. They remain separate in their course through the ganglion, lying immediately beneath its dorsal surface, and give off from the outer margins the lateral nerves, which, like themselves, leave the ganglion by constricted apertures in its neurilemma, and are accompanied by granular matter.

The tubular appendages of the ganglion-corpuscles of the first kind are all directed towards the exit of the nerves, and become connected with them near or at the point at which they emerge. From their attachment to the corpuscles, the appendages gradually become narrow, till they attain a certain permanent thickness nearly equal to that of the primitive nervous fibres into which they are continued.

* Edinburgh Medical and Surgical Journal, Jan. 1844. † Report, 1842, p. 36.

‡ Müller's Archiv. 1844, Heft ii, p. 76. His account is very similar to that of Helmholtz, who, in his dissertation 'De fabricâ syst. nervosi evertebratorum,' Berol. 1842, (analysed in Müller's Archiv. 1844, Jahresber,) has described the like structure in many more species, and especially the direct passage of the nerve-fibres into the ganglion-corpuscles of the cephalic ganglion of the leech.

|| The chief subjects of examination were the leech, Helix pomatia, Astacus fluviatilis, and Lymnæus stagnalis. Details of the modes of preparing the nerves for examination are fully given in the paper.

*Peripheral terminations of the nerve-fibres.* The investigations of Henle and Kölliker,* have proved a new and peculiar mode of termination of the nerve-fibres in the little bodies, seated especially in the nerves of the fingers and toes, which were discovered and to a certain point well described by Pacini of Padua, in 1830. These bodies (to which the name of *Pacinian corpuscles*† is now given,) are found in man at all ages after the twenty-second week of fœtal life, and under all circumstances, and in many mammalia. They are most numerous on the cutaneous nerves of the hands and feet; but they occur also sometimes on other sensitive cerebro-spinal nerves, and on the sympathetic plexuses in the mesentery and mesocolon, and about the pancreas; where they are especially numerous in cats. In man, from 150 to 350 may be counted on a single limb: and they are chiefly abundant on the branches of the digital nerves just penetrating the cutis; to which they are attached singly, or in pairs, or, sometimes in groups, by little fibro-cellular pedicles. Through the pedicle of each, a single primitive nerve-fibril passes into the corpuscle.

The corpuscles are of various form, elliptic, ovate, obovate, crescentic, or reniform: they measure, (in parts of a line,) from ·66 to 1·2 in length, and from ·45 to ·6 in breadth. They are semitransparent, slightly glistening, and appear as if a central cord passed through them. Each of them is composed of from 40 to 60 very thin coats, arranged round a central canal or cavity, like so many capsules enclosed one within another; and each coat or capsule is composed of two layers of fibro-cellular tissue, an outer layer with circular, and an inner with longitudinal fibres. Between each two adjacent layers or capsules, there is an albuminous fluid; it is most abundant between the outer capsules, which are less compactly arranged than the central ones. The outermost of all the capsules in each corpuscle is connected by cellular tissue with the adjacent parts, from which also blood-vessels penetrate inwards through more than half the layers. Here and there the adjacent capsules appear connected by partial septa extending across the spaces containing the fluid, and this is especially the case at the end opposite to the pedicle. The canal or cavity in the axis of each corpuscle contains a fluid like that between the capsules, and, in this fluid, a primitive nerve-fibril. The nerve-fibril after traversing the pedicle of the corpuscle, and a conical prolongation from the end of the pedicle through the substance of the lower part of the corpuscle, enters the cavity, and at once becomes smaller, paler, and flatter. It passes along the cavity, and at or near its distal end, terminates in a knob, or by bifurcating: in no case is anything formed like the *terminal loops* of nerves, and it is very rarely that more than one nervous fibril enters a corpuscle; neither does the terminal enlargement of the nerve-fibril resemble a ganglion corpuscle.

Of the use of these bodies little can be said. It is suggested that as their construction with alternate layers of membrane and fluid is rather like that of the electric organs of the electric ray, &c., these also may be electric organs, and, according to Pacini, the chief agents in mesmeric operations. But Henle and Kölliker could find no manifestations of free electricity in them during life. Their not occurring upon any known motor nerves, would appear to prove that they have nothing to do with motion; but their existence on many nerves of the sympathetic system, and their non-existence on many sensitive nerves, make it also probable that they are not connected with acuteness of sensation. They may be electric organs, as their peculiar structure suggests; but before they can be concluded to have any relation to animal magnetism, it would be advisable to prove that *that* has any relation (except in name,) to physical magnetism or any form of electricity.

* Ueber die Pacinischen Körperchen; Zurich, 1844, 4to.

† A minute account of them will be found in the last January Part of this Journal. A further account of them in several mammalia is given by Pacini, in the Annali Univer. di Medicina, Gennaio, 1844. They are described as lacteal organs by M. Lacauchie, the nerve-fibril being taken for a chyle vessel, in his communication to the French Academy of Sciences, Oct. 30, 1843; and, more recently, by Mayer, as glands with excretory ducts passing into the nerves, in the Oesterr. Med. Wochenschrift, Sep. 14, 1844; from the Med. Corresp. Rhein. und Westphal. Aerzte, No. 3, 1844.

GENERAL PHYSIOLOGY OF THE NERVOUS SYSTEM.

The publication of the collected observations of MM. Matteucci and Savi is an important fact in this year's history of the progress of nerve physiology; but the contents of their work* are not of this year's growth, consisting as they do, almost entirely, of the observations which have been a long time in course of publication,† and are now collected and arranged. They cannot therefore be brought within the range of this Report; though the observations which M. Matteucci has made since the publication of his work properly fall within it.

Of these continued observations, some were made with M. Longet‡ to determine the influence of electric currents on the anterior roots of the spinal nerves and the anterior and lateral columns of the cord. The result was that their influence is wholly different when exercised on centrifugal fibres alone, from what it is when exercised on mixed fibres. In the latter case, (according to the experiments performed before these last,) contractions take place only at the commencement of a current directed centrifugally, and at the interruption of a current directed centripetally; in the former case the contractions ensue only at the closure of the circle with a centripetal current, and the opening of one with a centrifugal current. The anterior (and, though with less energy, the lateral) columns of the cord act, in these respects, like the motor nerve fibres.

Other researches§ show that there is a direct proportion between the quantity of electricity employed in repeatedly exciting (through the lumbar nerves,) muscular contractions in the posterior extremities of frogs, and the amount of force exerted by the muscles thus contracting. The proportionate quantities of electricity were measured by the quantity of zinc which was dissolved in its production in the several experiments; and the muscular force by the distance through which a weight attached to the feet was drawn. The muscular force was reduced to one half and to one third, when the current was reduced, in different experiments, to those amounts. The amount of force exercised by the muscles was six times as great as would be obtained by the combustion of the same quantity of zinc, or by using the same current in an electro-magnetic apparatus.‖

In an endeavour to establish a theory that the cerebro-spinal nerves (excepting nerves of special sense,) should be classed as muscular and cutaneous nerves, and not as motor and sensitive, Dr. J. W. Arnold¶ (Heidelberg,) relates some experiments which render it possible that the mind derives the consciousness of the position of a muscle at any given time through the medium of the motor, and not of the sensitive nerves, or at least through the medium of filaments which are included in the anterior spinal roots. The chief fact is that when the posterior roots of the nerves of the posterior extremities of a frog are divided, although no external stimulus of either hind-leg excites movements of it, yet when by exciting other and sensitive parts, the frog is induced to move its hind-legs, it always first puts them into a position adapted for the performance of the intended movement. *E.g.*, if one of the hind-legs of a frog has had its sensitive (posterior) nerve-roots cut, and this leg be extended, when the frog wishes to leap, it first draws this leg up, and then leaps with it as well as with the others, all the nerves of which are entire; as if, though the leg could convey no sensation of objects, it still was able to give the subjective sensation of the position of its muscles.

[The fact is singular, but far from sufficient to prove the theory. It is contradicted also, so far as man is concerned, by the cases of persons who having lost the

* Traité des Phénomènes electro-physiologiques des Animaux; Paris, 1844, 8vo.

† In the Bibliothèque Universelle de Génève; whence extracts have been commonly published in other journals.

‡ Gazette Médicale, 14 Sept. 1844. Report from the Acad. des Sciences, 9 Sept. 1844.

§ Gazette Médicale, 21 Sept. 1844. Report from the Academie des Sciences, 16 Sept.; and Annales de Chimie et de Physique, Août, 1844.

‖ There are further observations on this subject in the just-published third part of the 2d volume of Valentin's Lehrbuch der Physiologie, of which a report will have to be given next year.

¶ Ueber die Verrichtung der Wurzelnder Rückenmarksnerven; Heidelberg, 1844, 8vo. Analysis in Schmidt's Jahrbucher, April 1, 1844. Mr. Swan appears also to think that the sensitive nerves are not the conductors of our impressions of muscular fibre. "The sensory judges of slight changes produced by motion on the skin;" but the forces of "motive action are appreciated by a specific centre, probably by the striated body;" "The principal offices of the brain, &c." 1844, p. 11.

sensation, but not the voluntary motion of the arms, are so unaware of the position and state of their muscles, that they are obliged to look at what they are holding, lest they should let them fall. The other part of the theory, namely, that those commonly called sensitive nerves should be called cutaneous nerves, is founded on the notion that when the skin is stripped off a frog's hind-leg, the limb is in a state similar to one of which the posterior nerve-roots are divided. Doubtless in such a state the frog does not willingly move its leg; but is the leg therefore insensible ?]

To prove the *functional independence* of the sympathetic nervous system, that is, that it is independent of, and essentially different from, the cerebro-spinal system in the discharge of its functions, Volkmann and Bidder* have published an extensive series of experiments on the effects of removing from frogs the brain, or spinal cord, or both, leaving in every case the medulla oblongata so that the respiratory movements might continue. The general result was to show the strongest contrast between the effects of the destruction in the parts supplied by cerebro-spinal nerves, and its effects on those supplied by the sympathetic. In the former, all the muscles were rendered at once incapable of contracting upon either voluntary or reflex stimulus; in the latter, it was long before any effect was produced. The circulation in the web continued unimpaired two weeks after crushing the cord, fourteen days after destruction of the brain, and five days after destroying both at the same time ; and the pulsations were as frequent and vigorous as in healthy frogs. Sufficient evidence was also afforded, that whether the brain or cord or both were removed, the processes of exudation and absorption were very well carried on in the limbs. [The contrary results obtained by Valentin and Stilling, are shown to have been due to the improper mode in which they kept the frogs.] The intestinal canal also continued, to the time of death, to be active and irritable, and both it and the heart remained capable of being excited to healthy movements for some time after the voluntary muscles had ceased to be excitable by any kind of stimulus. Urine also was secreted in natural quantity, but was retained in the bladder, which by the destruction of the central parts of its cerebro-spinal nerves had lost its power of contraction. Food also was digested completely, and in the ordinary period, in the stomach ; so that, on the whole, no organic function was materially disturbed by the destruction of the brain and spinal cord, although, by the integrity of the medulla oblongata and the respiration, the animal lived many days. Since during the same time all automatic and other movements really dependent on the cerebro-spinal centres ceased, it is deduced [and with great probability, at least, in frogs,] that the functions of the sympathetic nerve are independent of its connexions with the brain and spinal cord.†

The experiments just cited regarding the secretion of urine after the loss of influence from the brain and cord, are corroborated by M. Segalas.‡ His experiments, and the cases of two patients recorded by him, tend to prove (against the experiments of Krimer,) that division or complete destruction of the lower part of the cervical region of the spinal cord, and of any or all parts below it, does not check the secretion of urine ; and that destruction of the upper part of the cervical portion does not affect it, provided artifical respiration be completely and early established. When the urine of numerous rabbits, guinea-pigs and dogs, secreted

* Müller's Archiv. 1844, Heft iv, p. 359.

† The paper contains many experiments indirectly bearing on the subject, and notices of the diseases of experimental frogs by which perhaps many failures of researches may be explained. As to the conclusions drawn from it,—it may show that the sympathetic nerves and ganglia will exercise their wonted influence upon nutrition, secretion, &c., long after the separation of their connexion with the cerebro-spinal nervous centres, but the facts do not prove that the influence of the sympathetic, on the parts in which its nerves are distributed, is different essentially from that exercised by the cerebro-spinal system on the parts which it supplies. They afford no evidence that, for example, an influence is exercised on nutrition or secretion, in one part of the body, through sympathetic fibres, more or otherwise than it is exercised in another part, through cerebro-spinal fibres; the connexion, also, which exists between the two systems and the sensations and movements of the parts in which they are severally chiefly distributed, differs in degree, but not, so far as yet is proved, in kind.

‡ Bull.de l'Acad. de Médecine, Sept. 15-30, 1834; and Medical Times, Sept. and Oct. 1844.

after such division or destruction, was analysed, it was found sometimes of healthy constitution, and when altered, the alterations were slight and not uniform. M. Segalas, therefore, deduces that the serious changes observed in the urine of men at a late period after injuries of the spine, are due to the products of inflammation excited in the coats of the bladder, either by its long-continued distension, or by the irritation of catheters kept in it. He further shows by experiments, that the secretion of semen is not directly affected either in quantity or in composition, by injury or division of the spinal cord.

### ANATOMY AND PHYSIOLOGY OF THE NERVOUS CENTRES.*

*Spinal cord.* In his description of the spinal cord and its membranes, M. Foville describes the following structures, none of which have, I believe, been hitherto noticed. 1. The existence of a thin external cortical layer surrounding the whole cord, formed by the more intimate approximation of the fibres. It is this which being penetrated by the roots of the nerves makes them appear as if they arose from a groove.† 2. Cords, rather less white and firmer than the rest of the white substance, extending all down the spinal cord, at the junctions of the central angles of its anterior and posterior columns with the margin of the corresponding commissure.‡ 3. The continuation of the posterior-internal (posterior pyramidal) tract, as low down as the lumbar enlargement of the cord.§ 4. The similar continuation of the thin lateral tract (that which is seen on the medulla oblongata, just in front of the restiform body,) down the whole length of the cord. 5. The arrangement of the longitudinal fibres of the cord in lamellæ whose edges radiate to and from the central axis of the cord, and which, by their connexion with the gray matter, may be said to be all fixed to the wall of its central ventricle.|| 6. The decussation of the anterior tracts through the whole length of the anterior commissure. 7. The mode of this decussation and of that at and above the anterior pyramids, by the layers or fibres passing not only from side to side, but from before backwards, so that those (for example,) from the right side become, in successive layers, the lateral parts of the gray matter of the left side.¶ 8. Prolongations of pia mater passing in from various parts of the surface of the spinal cord, and converging to its centre.** 9. The commencement of the lumbar enlargement of the cord by an elongated narrow eminence, (like an olivary body,) at the antero-lateral angle of its lower portion.††

Dr. Budge‡‡ has examined the connexion between the spinal nerve-roots and the cord of frogs, and wholly opposes his observations to those of Stilling and Wallich, who he believes have been deceived by using too low microscopic powers, and by not observing the changes of direction of the nerve-filaments at the torn or cut portion of the cord. He traces the filaments of the nerve-roots passing straight upwards in the cord, those of the upper roots overlaying those of the lower, and all becoming rather smaller. He believes that there are no longitudinal fibres in the cord which are not continuations of the nerve-fibres. Transverse fibres are found in it, but are obscure; he considers many of them to be fibres connecting (like bridges,) the ganglion globules, or their membranous en-

* On the comparative weights of the several parts of the nervous centres, see M. Bourgery's communication to the Acad. des Sciences, 23 Sept. 1844, reported fully in the Gazette Médicale, 5 Oct.; and, for many interesting considerations on their several functions, see Mr. Swan's work, "The principal offices of the brain and other centres," London, 8vo, 1844. The principal part of the contents of M. Foville's great work, 'Traité complet de l'Anatomie......du Système nerveux cérébro-spinal,' 1re partie, Paris, 1844, 8vo, were published before this year in his article *Encéphale* in the Dict. de Méd. et de Chir. pratique, and in the reports on his memoirs on the structure of the brain and skull, made to the Paris Academy of Sciences by M. de Blainville in June 1828 and May 1840; and to the Paris Academy of Medicine in the latter year by M. Blandin. I have therefore reported nothing but those views of M. Foville which are, I believe, peculiar to him, and which appeared first in the volume lately published; they relate exclusively to the structure of the spinal cord and the origins of nerves.

† L. c. pp. 136, 282, &c. ‡ L. c. pp. 284-5. § L. c. p. 283, &c.

|| L. c. p. 291. ¶ L. c. pp. 294-6, 318, 324, &c.

** L. c. p. 342. These must be, or contain, the vessels injected by Mr. Smee, and described by Dr. Todd, (Cycl. of Anatomy, art. Nervous System, p. 708.) †† L. c. p. 138.

‡‡ Müller's Archiv. 1844, Heft ii. The examinations appear to have been carefully made; the greater part of the long paper is a detail of the precautions to ensure accuracy.

velopes; but is doubtful whether some are not independent transverse nerve-fibres. He was unable to trace any of the nerve-fibres to the brain.

[These results are directly opposed to those experiments of Van Deen,* quoted in the last Report, which seemed to prove that the nervous fibres do not proceed to the brain, but terminate in the spinal cord; for impressions did not appear to pass much, if at all, beyond the part of the cord on which the several nerves irritated were connected with it. The results of some experiments which I made with Dr. Baly upon turtles were as contrary to the conclusions of Van Deen as these examinations by Budge are. All the spinal roots of both sides, in the cervical and superior dorsal regions, were divided so that a portion of the cord six inches long had no nerves connected with its sides. Its connexion with the lower part of the cord being unimpaired, I found that every irritation of the upper end, or any other part of this loose portion of the cord, produced vivid movements in the hind limbs and tail. The movements were perhaps rather more vivid when the posterior columns were irritated, so as to produce them by reflex influence, than when they were excited directly by irritation of the anterior columns. I very gently cut slices from the cord (as in Van Deen's experiments,) and at every cut the movements of the hinder parts were produced.]

Dr. Poletti,‡ unaware of the experiments which had already been made by Dr. Engelhardt, has observed the effects produced on the posture of the limbs of frogs, by dividing (not crushing, as in Engelhardt's experiments,) the spinal cord in different parts. When it is divided just below the occiput, the limbs are permanently flexed; the flexion becomes less decided the lower the cord is divided, till the division comes to the level of the space between the fourth and fifth vertebræ. When the cord is divided below this point, the limbs which before were flexed, are sometimes suddenly forcibly extended; they remain for some time fixed parallel to one another and to the axis of the trunk, and then gradually relax.

*Brain.*§ An excellent paper on the structure of the cerebellum has been published by Dr. C. Handfield Jones,‖ in which many parts of Reil's obscure description are explained or rectified. The sum of his account (so far as it may be made intelligible without diagrams,) is as follows: the medullary or fibrous nucleus of the cerebellum has not (as Reil and Foville suppose,) a separate "exterior shell," or "laminated stratum," from which alone the medullary axes or stems of the lobules are derived, but consists throughout of similar medullary plates, which decussate with each other as they pass onwards from the base or junction of the crura, and which are all destined to pass into the lobules. The central medullary stem of each lobe or lobule consists, first and mainly, of the medullary plates which are given off in succession from the exterior of the medullary nucleus of the hemispheres, or the lobe, as the case may be. But, besides these, the stem of each lobe or lobule has, 2dly, (what are, probably, a part of that which Foville describes as the white nervous lining of the cortical substance,) commissural plates which pass to and from the lobe or lobule next to it on each side. The first of those sets of medullary plates, which lie in the middle of the stem of each lobule, are not (as Reil supposed them) derived from the *ridge* on which that very lobule stands, and with which the *furrow* at its base forms the *articulation;* but they are those plates which form the highest part of the ridge of the lobule next preceding it. The same may be said of the lobes and their ridges. The ridge below the base of each lobule is thus formed by the elevation of those plates of the stem of the lobe which will compose the greater part of the stem of the next following lobule; and the furrow at the base of each lobule is bounded at its *proximal* side [*i. e.* the side at which the plates or fibres, if traced from the greater to the less

* Related in the Tijdschrift voor naturliche Geschiedeniss en Physiologie, 1842, D. ix.

‡ Il filiatre sebezio, Dec. 1843.

§ An account of numerous careful repetitions of Magendie's and other experiments on the pulse-like movements of the brain and spinal cord has been published by Dr. Alex. Ecker, in his 'Physiol. Unters. über die Bewegungen des Gehirns und Rückenmarkes; Stuttgart, 8vo, 1843. His conclusions fully confirm those of Magendie; especially as regards that of the respiratory movements of the brain being mainly due to the *ascent* and *descent* of the cerebro-spinal subarachnoid fluid as it is alternately subjected to and freed from the pressure of the vertebral sinuses and other veins, which become alternately full and empty in the acts of expiration and of inspiration.

‖ London Medical Gazette, March 29, 1844.

divisions of the cerebellum would first arrive,] by the plates given to its medullary stem from the stem of the lobe, and in its distal side by the commissural plates going from itself to the lobule next beyond it.

The same plan of arrangement is repeated in the construction of the several parts of a lobule. As its medullary stem passes along its centre, it gives off in succession subordinate plates which pass into the middle of each lamina or subordinate lobule; those which go to the lamina on the *distal* side of the lobule having decussated across the fissure in its axis. And besides these plates which form the axis of the stem of each lamina, each has also, like each lobule, commissural plates by which its medullary stem is connected with those of the laminæ next before and after (or above and below) it. Moreover, as with the lobules, so with the laminæ, the plates of the medullary stem of each are not those which form its own ridge, but those which formed the ridge of the lamina next preceding it, and the plates which form its ridge are those which will pass into the central stem of the lamina next beyond it.

### ANATOMY AND PHYSIOLOGY OF THE NERVES.

*Olfactory nerve.* M. Foville* maintains that nearly all the deep origins that have ever been assigned by different anatomists to this nerve, do really belong to it; and that it is attached to the gray matter of the convolutions, the band of the convolution of the corpus callosum, the anterior crura of the fornix, the surface of the locus perforatus anticus, the fibres continued from the posterior tract of the medulla oblongata into this *locus*, the external portion of the gray matter of the corpus striatum, and the fibrous layer which invests it; and, lastly, to the anterior commissure. [But he does not appear to have direct evidence of all these origins in the human subject.]

*Optic nerve.* M. Foville† rightly describes the connexion between the commissure of the optic nerves and the lamina cinerea as being more intimate than, since it was described by Vicq. d'Azyr, it has been generally considered. He describes the gray matter at this part as the *anterior gray root* (the posterior being that commonly known, from the infundibulum); it is connected with the anterior pillars of the fornix; it is covered by a very thin white layer which extends from the commissure over the locus perforatus to the superficial layer of the adjacent convolutions; and some of the gray matter passes into the substance of the nerves. He says also that the continuation of the optic tract is not only spread over the whole upper surface of the thalamus and corpora quadrigemina, but is connected with the direct median and external prolongations of the posterior tract of the medulla oblongata with the tænia semicircularis, and by a "little nervous membrane (?)" with the temporal tuberosity of the convolution of the corpus callosum.

*Fifth nerve.*‡ The large sensitive portion of this nerve is said by M. Foville§ to pass obliquely to the outer edge of the restiform body through part of the substance of the middle crus cerebelli. From the posterior edge of this prolongation of its roots a nervous membrane passes into the nucleus of the cerebellum; and from its anterior edge fibres pass which form some of the transverse arches of the pons, or are sometimes continued into the transverse fasciculi on the floor of the fourth ventricle, where, as well as in the nervous expansion which he supposes to line the cortical substance of the cerebellum, these roots are closely connected with those of the auditory nerve.

The roots of the small motor portion are traceable, he thinks,‖ to those fibres of the lateral tract of the medulla oblongata which are given from it to the middle crus cerebelli.

*Auditory nerve.* To this, also, M. Foville¶ assigns an origin not less complex

* Traité, &c. pp. 518, 525. † L. c. pp. 182, 510, &c.

‡ Some facts relating to the branches of the fifth and facial supposed to supply the muscles of the paate, will be found in the following account of the experiments of Dr. Hein.

§ L. c. p. 507. ‖ L. c. p. 531. ¶ L. c. p. 505, &c.

than those of the nerves already mentioned. He says that a fine white membranous nervous tissue is prolonged from its roots, from those of the fifth, and from the surface of the restiform body; which tissue, after surrounding the crus cerebelli [in which it is not difficult to see it], forms a lining (*doublure*) to the whole of the cortical substance of the cerebellum. Another nervous membrane, continuous with the preceding, lines the whole of the walls of the fourth ventricle, and is continued into some of the transverse striæ; and others are combined with the medullary velum and pass to the corpus dentatum. Lastly, other emanations from these roots pass forwards and inwards, and are confounded with the transverse arches of the pons.

*Facial nerve.* Dr. Hargrave* has related a case in which, as in that by M. Diday, mentioned in the last Report, the uvula was drawn to the left side in paralysis of the facial nerve, and recovered its median position when the disease ceased. [Both this case, and one of a similar kind by Dr. Williams,† afford additional evidence for believing that the facial nerve is the motor nerve of the levator palati and azygos uvulæ muscles, and sends its fibres to them through the superficial petrosal branch of the vidian nerve and the spheno-palatine ganglion. And this opinion must, I think, be retained concerning the arrangement of these nerves in men, although apparently contradicted by the experiments of Hein.‡] He could never in dogs, calves, and goats, produce contraction of any of the muscles of the palate by irritating the root of the facial nerve, neither was the contraction which he produced by irritating other roots affected by destroying the petrosal nerves going to the spheno-palatine ganglion. He succeeded, indeed, in tracing a branch of the middle posterior palatine nerve (through which the petrous filaments of the facial are supposed to pass after traversing the spheno-palatine ganglion,) into the substance of the tensor palati and azygos uvulæ: but this branch enters the former muscle at its tendinous part, while that of the pneumo-gastric and accessory, which his experiments indicate to be the real motor nerves of these muscles, enters the tensor in its belly.

*Nerves of the eighth pair.* Among the nerves most examined during the last year are the glosso-pharyngeal, pneumo-gastric, and accessory; on the offices of which observations have been lately made by Van Kempen,§ Krause,|| Bernard,¶ Bischoff,** and Hein.†† The chief questions to be determined were—1st. Whether each or all of these nerves be composed at their origin of wholly sensitive, or wholly motor, or mixed, fibres? 2d. What muscles derive their motor nerves from each or either of them? The present state of the facts is as follows.

1. The *glosso-pharyngeal nerve* is, by general consent, chiefly sensitive; having filaments for both taste and common sensation. But it is probably, also, a motor nerve. Panizza, John Reid,‡‡ and Longet,§§ have maintained that it is not: having failed to discern movements in any muscle when irritating its roots within the skull, and referring the movements produced by irritating it outside the skull to filaments of other nerves mixed with it, or to reflex acts following the impressions conveyed through its centripetal fibres to the medulla oblongata. But other and more certain experiments are in favour of its having a direct motor influence. These include the experiments of Müller, |||| Volkmann,¶¶ and Hein. The last-named, whose experiments were very carefully performed, states that his results completely agree with those of Volkmann. When the roots of the glosso-pharyngeal nerve were irritated in the recently cut-off heads of calves and dogs, after removing the brain and medulla oblongata, and separating these roots

* Dublin Medical Press, Dec. 6, 1843. † Ibid. March 5, 1844.

‡ Müller's Archiv. 1844, Heft iii-iv. See also his account of the nerves of the palate, p. 583.

§ Essai experim. sur la nature fonctionnelle du nerf pneumogastrique; Louvain, 1842, 8vo; analysed by Bischoff in Müller's Archiv. 1843, Heft vi.

|| Handbuch der menschl. Anatomie, Bd. ii, 1843. ¶ Arch. Gén. de Méd., Avril et Mai, 1844.

** Müller's Archiv. 1843, Jahresbericht, p. clv.

†† Ib. 1844, Heft iii, iv, pp. 297-358; from an inaugural dissertation, Heidelberg, 1843.

‡‡ Experimental Inquiry, &c.; Edinburgh Medical and Surgical Journal, 1838, vol. xlix.

§§ Anat. et Phys. du Système Nerveux, t. ii, p. 220.

|||| Physiologie des Menschen, Bd. i, p. 639, Ed. i.

¶¶ Ueber die motor.-Wirkungen der Kopf-und Hals-nerven. Müller's Archiv. 1840, p. 475.

from those of the pneumo-gastric, contractions always ensued in the stylo-pharyngeus muscle. He believes, also, that the terminal branches of the glosso-pharyngeal give motor filaments to the palato-glossus muscle, although he could not make it contract by stimulating either this or any other nerve; for glosso-pharyngeal filaments alone can be traced to this muscle, and its not contracting may be due to the disturbance of connexions by the dissections necessary for the experiment.

2. *Pneumo-gastric and accessory nerves.* The pneumo-gastric nerve is, by general consent, chiefly sensitive: but it is questioned whether the motor fibres, which many of its apparent branches contain, are derived directly from some of its own roots, or from those of the accessory, through its internal branch, which unites with the trunk of the pneumo-gastric soon after the formation of the jugular ganglion. The opinion that the roots of the pneumo-gastric are wholly sensitive, and those of the accessory wholly motor, and that the two bear to each other the same relation as the anterior and posterior roots of the spinal nerves, has been especially maintained by Valentin,* Longet,† and Morganti,‡ following in the steps of Scarpa, Arnold, and Bischoff. But the experiments of all others, and especially those of more recent date, though they agree in few things besides, are all opposed to this opinion. Among these are the experiments of Müller,§ Volkmann,‖ John Reid,¶ Stilling,** Van Kempen,†† Bernard,‡‡ and Hein,§§ of which those of the last four must be included in the history of this year.

Stilling's experiments tend to show—1st, That irritation of the roots of the pneumo-gastric produces movements in the pharynx, glottis, stomach, (and heart): 2d, That irritation of those of the accessory nerve produces no movement in any of these parts: and 3dly, That irritation of the trunk of the pneumo-gastric, and of the superior and inferior laryngeal nerves, produces movements in the pharynx. He concludes from these and others that the fibres of the superior laryngeal nerve are (as to the larynx) wholly centripetal; those of the inferior laryngeal mixed, giving centripetal fibres to the trachea, and motor fibres to all the muscles of the larynx; and that the fibres of the internal branch of the accessory which joins the vagus are all centripetal.

Van Kempen's experiments on the roots of the pneumo-gastric and accessory agree with Stilling's, in showing that in the roots of the pneumo-gastric there are both sensitive and motor fibres; and that the accessory nerves give motor fibres to no muscles besides the sternomastoid and the trapezius. He concludes also that the pneumo-gastric nerve is the motor nerve of the constrictors of the pharynx, the palato-pharyngeus, the œsophagus, and the interior muscles of the larynx; and that irritation of its roots, as well as of those of the accessory, is wholly without influence on the movements of either the heart or the stomach. But he differs from Stilling, and agrees with all others, in holding that the superior laryngeal contains a few motor fibres, and that with these it supplies the crico-thyroideus muscle.

On the other hand, many others are agreed, against Van Kempen, that irritation of the pneumo-gastric does produce movements of the stomach, at least while digestion is going on; and in affirmation of this, Longet's experiments appear conclusive.¶¶ And, as to the influence of the pneumo-gastric on the muscles of the palate, both Hein's experiments, presently to be mentioned, and Bischoff's,*** agree that in domestic animals irritation of its roots produces movements not only of the palato-pharyngeus, but also of the levator palati and azygos uvulæ.

The experiments of M. Claude Bernard, on the roots of the pneumo-gastric and accessory, were made by seizing the accessory at its exit from the skull, and plucking it out by its roots.

* De functionibus nervorum, p. 45.

† L. c. t. ii, p. 262, e. s.

‡ See last Report, and Archives Gén. de Médecine, Novembre 1843, where his experiments are fully detailed.

§ Physiologie, Bd. i, p. 641.

‖ L. c.

¶ L. c.

** Haeser's Archiv. Heft iii, 1842; and Report of the Scientific Meeting at Brunswick, in Müller's Archiv. 1843, Heft vi; Bischoff's Jahresbericht, p. cliv.

†† L. c.

‡‡ L. c.

§§ L. c.

‖‖ Bidder, in Müller's Archiv. Heft iv, 1844.

¶¶ L. c. p. 322. They are lately confirmed by Bischoff, Müller's Archiv. 1843, Jahresber. p. clv; and by Stilling and Valentin, as far as the *fact*, but not the explanation, of the movements is concerned.

*** L. c.

This mode of experimenting has the advantage of not rapidly destroying life; the animals lived for many days or weeks, till they were killed for the sake of the autopsy. But it is, even more than the others, subject to fallacy, in consequence of the frequent mingling of the fibres of the pneumo-gastric and accessory, before they leave the skull, as well, perhaps, as in consequence of the injury that may be done to the medulla oblongata.

The results of these experiments were—1st. That all the roots of the accessory are insensible, except those which arise from the medulla oblongata; these appear to have "a certain dose" of sensibility, and to have the more the nearer they are to the origin of the pneumo-gastric. The external branch of the nerve, which, with Morganti and others, he considers to be formed exclusively from its lower roots, is wholly insensible, till it has mingled with filaments of the cervical nerves. 2d. It is only from the upper roots of the accessory nerve that its laryngeal filaments are derived; when they are destroyed the voice is completely lost; when they remain the voice is not affected by the destruction of all the lower roots. 3d. The effects of the complete destruction of the roots of the accessory on both sides are, complete loss of voice, some difficulty of deglutition, shortness and interruption of expiration when the animal tries to cry out, panting during great movements and efforts, and sometimes irregularity of gait: but when the animal is at rest, respiration, circulation, and digestion are performed as perfectly as in health. 4th. It is hence evident that the accessory nerves do not furnish all the motor filaments to those parts to which the branches of the pneumo-gastric nerves are distributed, and it is made probable that they furnish very few, if any, filaments to the organs below the upper part of the œsophagus.

[In Longet's experiments of galvanizing the trunk of the accessory no movements of the stomach, trachea, bronchi, or heart were ever seen; and M. Bernard justly observes, that it is hardly imaginable that so small a branch as the internal branch of the accessory should supply filaments to be distributed in all the parts to which motor influences are conveyed through the branches of the pneumo-gastric.]

5th. It is, further, made nearly certain that the accessory nerves do not supply all the motor filaments of the larynx and pharynx. For, as regards the larynx, the effects of the division or destruction of the accessory nerves, are very different from those of dividing the trunks of the pneumo-gastric nerves below their junction with the internal branches of the accessory. The latter operation completely paralyses the muscles of the larynx, and young animals die at once suffocated by the closure of the glottis; by the former, the voice is destroyed, but quiet respiration is performed with ease, and when the larynx is exposed, the glottis, though it remains widely open and is nearly immovable, yet moves as much as it does in ordinary quiet respiration, and the animal's life is hardly endangered. 6th. It is not experimentally determined what largyngeal muscles, besides the crico-thyroid, in which paralysis was proved, are paralysed by destroying the accessory; but the power of closing the glottis is lost, and vocalization is impossible. Consequently, since the power of keeping the glottis open against the influence of the atmospheric pressure in respiration, is not lost, (as it is when the pneumo-gastric are divided,) it is presumable that the arytenoid, the thyro-arytenoid, and the lateral crico-arytenoid are also more or less paralysed, while the posterior crico-arytenoid retain their power.* 7th. With regard to the movements of the pharynx, the animals which had lost their accessory nerves, after eating, perhaps, rapidly for a time, were obliged to eat slowly, stopping and raising the head at each effort of deglutition; and if they were hurried, portions of food often passed into the larynx. [This may most probably be ascribed to the partial paralysis of the

* M. Bernard strangely misinterprets his facts to support an hypothesis that the accessory nerves are the special *vocal*, and the pneumo-gastric the *respiratory* nerves of the larynx. But coughing is no vocal act; yet it is impossible when the accessory nerves alone are divided, because the closure of the glottis is not possible. M. Bernard says nothing definite about coughing, or any similar complex respiratory act; but, no doubt, the interrupted efforts of expiration of which he speaks were attempts at coughing, not recognised because the glottis was not closed and there was no explosive noise.

muscles of the pharynx, but M. Bernard supposes that as the larynx has, in his opinion, both a vocal and a respiratory motor nerve, so also the pharynx has some branches from the pneumo-gastric for the simple movements of deglutition, and some from the accessory nerves for those movements by which in deglutition the glottis is closed. The conclusion is improbable, and the experiments show that there were difficulties in deglutition, independent of the non-closure of the glottis.]

8th. By the division of the external branch alone of the accessory nerves no effect was produced on the voice or deglutition, but the animal was put into the condition of a *short-breathed* person; its cries became short and interrupted; it was quickly *blown* when it made violent muscular efforts, and when the respiration was hurried; there was sometimes imperfection in the movements of the fore limbs. Of these effects the shortness of the cry is ascribed, by M. Bernard, to the loss of power in the sterno-mastoid and trapezius muscles, by the contraction of which the descent of the sternum and upper ribs is for a time hindered in healthy vocal expiration. In like manner, the paralysis of these muscles (and of others dependent for fixed points on parts to which they are attached) hinders the fixing of the walls of the chest which is necessary for efforts. 9th. But the paralysis is not complete. M. Bernard relates an experiment in which, after division of the external branches of the right accessory nerve, both sterno-mastoid muscles contracted with equal force in forcible respiration, but the left alone contracted when the animal cried out.*

Hein's experiments relate almost exclusively to the nerves of the palate. They consisted in irritating the roots of the several nerves within the skull of an animal just beheaded, after removing the brain, so that there was very little danger of a part of one root being mistaken for another. His conclusions regarding the motor fibres of the glosso-pharyngeal are already mentioned. As to those of the pneumo-gastric and accessory nerves, they tend to show that these nerves send branches, through the medium of the pharyngeal branches of the glosso-pharyngeal, to the levator palati, azygos uvulæ, and palato-pharyngeus. These branches, near their distribution, are almost too minute for dissection; their existence was proved chiefly by the results of the experiments, which at last guided to the detection of them running just under the mucous membrane to the muscles. But he does not pretend to distinguish between the effects (on the muscles of the palate,) of irritating the pneumo-gastric and the accessory; he believes that their fibres are from the roots mingled, and that they both influence all the muscles supplied from the branches of either.

[From all these facts the first conclusion must be, I think, that the precise functions of these nerves are not yet determined. The most probable account may be—

1. That the glosso-pharyngeal is chiefly the nerve of the sense of taste, and, in a less degree, a nerve of common sensation.

2. That the glosso-pharyngeal is, according to the experiments of Müller and Hein, the motor nerve of the stylo-pharyngeus, and probably, also, of the palato-glossus. The branches which it gives to the digastricus, stylo-hyoideus, and constrictors of the pharynx, appear, according to the experiments, to be sensitive, or else derived from the facial and accessory nerves, with which it has previously united.

3. The pneumogastric is, from its origin, composed of both sensitive and motor fibres. But it cannot be decided at present, whether it alone supplies any particular muscles; or whether, in all its muscular branches, and especially in those given off above the œsophageal, there are filaments from the accessory as well as from its own roots.

4. The accessory nerve contains in all its lower roots motor fibres alone; in its upper roots it is not improbable that there are some sensitive fibres also. It is

* Hence he supposes that these muscles, and the trapezii, like those of the larynx, have a vocal and a common respiratory function, and a separate nerve for each; so that while they act as common inspiratory muscles under the influence of the cervical nerves, they arrest the respiratory acts when they are under the influence of the accessory nerves. He would, therefore, call these nerves the antagonist rather than the accessory nerves of respiration.

a motor nerve of the sterno-mastoid and trapezius muscles; and it is very probable that it gives, by its internal branch and other communications, motor fibres to the pneumo-gastric, from which they are subsequently distributed to some or all of the muscles of the larynx,* and pharynx; and, in some animals, to muscles of the palate.

It is evident that the chief difficulty is in distinguishing the functions of those roots which may be either the uppermost roots of the accessory, or the lowest of the pneumo-gastric. In the last Report an observation by Mr. Spence of a root which he considered to be the motor root of the pneumo-gastric was mentioned. That root had its origin midway between the pneumo-gastric and the accessory roots; and both Krause and Hein agree that it is not unfrequent to find that several of the upper roots of the accessory (as they are usually described,) are united to and interchange filaments at the jugular ganglion of the pneumo-gastric. In a preparation which I lately made, several of these minute roots are commingled with roots of the pneumo-gastric, even before the ganglion is formed on it. It is therefore difficult to say whether these are roots of the pneumo-gastric or of the accessory; and the more difficult, because the upper roots of the accessory arise more and more nearly from the posterior columns of the medulla oblongata; and, moreover, whatever these roots may be, such communications as I have observed might effect an exchange of filaments between the two nerves before their trunks are formed. It is probable, also, that there are differences in these points in different species, and in different individuals of each; so that before we can hope to distinguish precisely the physiological properties of these two nerves, we must learn to distinguish them (if indeed they are two nerves,) anatomically.]

*Inferior laryngeal nerve.* Mr. Jackson† has recorded a case of aneurism of the distal two thirds of the arch of the aorta, in which the left inferior laryngeal nerve was involved, and a firm manly voice was changed to a squeaking whisper. Severe paroxysms of dyspnœa ensued on exertion, and sometimes during swallowing—signs referable to paralysis of all the muscles of the left side of the larynx, except the crico-thyroidei and thyro-hyoidei, by the unopposed action of which the vocal ligament was kept unnaturally tense, while in violent inspiration the arytenoid cartilage was not hindered from being, as in Dr. J. Reid's experiments, forced down like a valve over the glottis.

Professor Barkow‡ (Breslau) describes a small ganglion, scarcely a line in diameter, on that terminal branch of the inferior laryngeal nerve, which after ascending over the posterior surface of the cricoid cartilage lies upon or among the fibres of the posterior arytenoid muscle. He calls it the arytenoid ganglion ulnar nerve.

Dr. Poletti§ finds that another branch of the deep palmar branch of the ulnar nerve, constantly exists in addition to those described by former anatomists. It is as large as the filaments given to the lumbricales muscles, and arises from the concavity of the arch which the nerve forms, opposite the lower margin of the os unciforme, or a little further on. After giving off a few secondary branches, when it is opposite the articulation, between the os unciforme and os magnum, it passes through the interosseous ligament, and goes to be distribnted in the synovial membrane of the metacarpal joints.

### ORGANS OF THE SPECIAL SENSES.

*Eye.* M. Maunoir,‖ of Geneva, to support the view which he has so long maintained of the existence of the sphincter muscle around the pupil, and the radiated muscle in the rest of the iris, states that galvanism excites contraction of both

* The arrangement of its internal branch in the chimpanzé, discovered by W. Vrolik, (see last Report,) is very important evidence for this view; and Bernard's experiments are not less so, for in them he could not destroy much more than the roots of the accessory, though he may sometimes have left some of them uninjured.

† Medical Gazette, Dec. 22, 1843.

‡ Report from the Académie des Sciences, Sèance du 26 Août, in the Gaz. Médicale, 31 Août 1844.

§ Il filiatre Sebezio, Dec. 1843. It is probably the same which Valentin describes as "filaments to the articulation." (Soemmering's Anatomie, Fr. edit. vol. iv, p. 517.)

‖ Report from the Académie des Sciences, 19 Fevr. 1844; in the Gazette Médicale, 24 Fevr.

these muscles, and that in a recent case of accident he had an opportunity of observing their antagonist action. A man received a small penetrating wound through the lower part of the cornea, and a point just higher up in the iris. When the cornea healed, a triangular false pupil was found at the wound of the iris. Both this and the natural pupil could contract and dilate; but, on the contact of light, the false pupil dilated and enlarged when the natural one contracted, and the false pupil contracted when, in the absence of light, the natural one was dilated. [The result of a number of observations in cases of artificial pupil, wounds of the iris, &c., by E. H. Weber,* went to prove, that the movements which occurred could not be explained by supposing that the iris has circular or radiated fibres, or both, but only by believing that its irritable part consists of fibres variously woven together without any definite direction.]

A good discussion of this subject has been published by Dr. C. R. Hall.† He considers that the iris has a circular muscle for the contraction of the pupil; that the dilatation of the pupil depends, probably, on the cellular tissue or the blood-vessels of the iris having an unusual vital contractility; and that the only effect of the elasticity of the tissues of the iris is to accommodate it to changes of size, and to restore it from extremes to a medium state—i. e. to the size which it usually has after death.

Valentin‡ has found, in two rabbits, that four months after the extraction of the lens, a new body has been produced smaller than it, yet presenting, to microscopic examination, the peculiar cells and fibres of the natural lens in stages of development similar to those through which they naturally pass. These were mixed, however, with minute granules; their arrangement was not quite regular in all parts; and in both cases there was at the lower part of the new lens, opposite the injured part of the capsule, a yellow turbid portion formed of a finely granular substance quite different from lens-substance. In both cases the capsule of the lens was transparent, without any trace of blood-vessels. The appearances indicated that the new lens was formed from cytoblastematous substance oozing into the emptied capsule, and that thus its harder central parts were those which had been longest engaged in development, and were most perfect. Ernst Brücke§ has pointed out an ingenious mode of demonstrating the structure of the vitreous humour, derived from the fact that when two solutions which will precipitate each other, permeate, or are imbibed in, membranous septa, the precipitate first forms in and upon the membrane. By placing the vitreous humour of an eye cut vertically and transversely behind the lens in a concentrated solution of acetate of lead, he found its surface soon covered with a white substance, and when, some hours after, a portion was cut off from the posterior part, the section was seen marked by fine milk-white streaks, which were edges of layers of which the outermost lay parallel to the retina, the innermost parallel to the posterior surface of the lens. The distances between the streaks were greatest in the axis of the eye, and became less towards the zonula, where they were about $\frac{1}{250}$ of an inch apart. The margin of the layer appeared to be connected with the hyaloid membrane near the zonula, but in what way could not be determined. The vitreous bodies thus treated with lead separate more easily in the direction of the layers than in any other.

*Eyelids.* Dr. Zeis‖ has found that the eyelids of young animals born blind (and probably, also, those of the human fetus in utero,) are held together by a thin layer of gelatinous substance, which is interposed between their adjacent margins, and bleeds when torn across very soon after birth. Every day after birth this layer (displayed by vertical sections through the eyelids,) becomes thinner; at the same time, the fissure between the eyelids grows deeper, making progress from before backwards, as if by the gradual eversion of the skin of their adjacent margins; and after the first day, vessels are not detected in the intermediate substance by either bleeding or minute injections.

* Hildebrandt's Anatcmie, Bd. iv, p. 80. † Edinb. Med. and Surg. Journal, July 1844.

‡ Oesterreichische medic. Wochenschrift, Fevr. 10, 1844; from Henle and Pfeuffer's Zeitschrift 1843, Bd. i, Heft ii.

§ Müller's Archiv. 1843, Heft iv, p. 344. Pappenheim (Specielle Gewebelehre des Auges, 1842, p. 182,) said that the vitreous humour, when treated by carbonate of potass, "may be stripped in concentric layers almost like a bulb;" but did not demonstrate any of these layers of membrane.

‖ V. Walther and V. Ammon's Journal für Chirurgie, Bd. ii, St. ii.

*Lachrymal gland.* M. Gosselin,* after numerous attempts to inject mercury into the lachrymal ducts, has made himself nearly sure that there are only two ducts which go to the principal mass of the gland, and that the other six or eight adjacent apertures in the conjunctiva are the orifices of ducts belonging to as many isolated small glands, which form what some have described under the name of the palpebral portion of the gland. Some only of these smaller glands appear to have ducts which open separately; the ducts of others open into one of the larger ducts. By the existence of these separate portions, M. Gosselin explains the fact that after removal of what was supposed to be the whole lachrymal gland, some secretion of tears has still continued, and the eye has not lost its brilliancy; tears being secreted by the remaining small glands.

*Tongue.* Mayer† has published many brief notes on the comparative anatomy of the tongue, from which he deduces that papilla exist on it, not in mammalia alone, but variously developed in birds, amphibia, and fish; and that the varieties of form which the papillæ present are due to the various arrangements of the same elementary clavate or fungiform shape. He thinks that all the papillæ in the human tongue may be organs of taste; and that those which are so chiefly are the smaller ones most thinly invested by epithelium. He says the hypoglossal nerve has often in man, and generally in horses, from one to three ganglia; and that in *many* mammalia it has a posterior ganglionic root.

STRUCTURE AND FUNCTIONS OF THE GENITAL ORGANS.

*Male genital organs.* In an excellent treatise by Dr. Kobelt,‡ the following descriptions are chiefly worthy of notice. 1. Before uniting with the other, each crus penis enlarges into a bulb-like swelling, the *bulbus corporis cavernosi penis.* The arteria profunda penis sends a special branch into this bulb; in which also, the divisions of this branch diverge, one backwards to the apex of the crus penis, the other forwards to anastomose with the trunk of the A. profunda penis. 2. The A. profundæ of the two sides form an anastomotic arch near the junction of the crura penis, and it is from the convexity of this arch that the A. corporis cavernosi of each side is given off. 3. Among the veins of the corpus cavernosum, several ascend from the groove between it and the anterior part of the C. spongiosum to the V. dorsalis penis; and others of the same kind, coming out from the groove nearer the pubes, ascend to the abdominal cutaneous veins. 4. The posterior part of the bulb of the C. spongiosum urethræ exhibits, more or less distinctly, two lateral hemispheres, (the representatives of the completely divided bulb of the marsupials,) which are separated by a narrow fibrous partition gradually ceasing as it proceeds forwards. And above and between these in the middle line it has a third lobe (*colliculus bulbi*), through which pass the membranous part of the urethra, the vessels of the bulb, Cowper's gland-ducts, &c. 5. The erectile tissue is continued from the bulb, in a less developed form, round the membranous and prostatic parts of the urethra into the neck of the bladder. It is well-developed in the caput gallinaginis, which probably serves, in its erect state, to prevent the reflux of semen into the bladder. 6. The artery which runs forward in the upper part of the corpus spongiosum to the glans is usually a branch of the pudic, not of the artery of the bulb. 7. Among the many veins proceeding from the C. spongiosum are a set which pass in two rows through its upper part, emerge from the groove between it and the C. cavernosum, run downwards and unite in a plexus with scrotal veins, from which their blood is carried to the inguinal cutaneous and other veins. 8. The fibres of the M. bulbo-cavernosus are in three sets. The superficial posterior three-fourths of them end in a tendinous lamella which insinuates itself between the crus penis and bulb, and then passing over the latter, unites with its fellow; so that this part of the muscle surrounds the bulb, and will compress it from below upwards, and from behind forwards. The anterior superficial fibres have a similar thin tendinous layer which winds over the side of the C. cavernosum, and unites with its fellow over the dorsal

* Arch. Gén. de Méd. Octobre 1843.

† Ueber die Zunge als Geschmacksorgan, in the Acad. Nat. Curios. vol. xx, p. 723.

‡ Die männlichen und weiblichen Wollustorgäne des Menschen, &c.; Freiburg, 4to, 1844.

vessels and nerves; and the fibres of this division will compress not only the anterior part of the bulb, but the body of the penis and its dorsal vessels. The third or deep set of fibres immediately cover and surround the hemispheres of the bulb, like a muscular hood, and form their proper compressor. 9. The M. bulbo-cavernosi serve especially to maintain and increase the turgescence of the glans both by retarding the current in its veins and accelerating that in its arteries. The arrangement just described enables their contracting fibres to compress all the veins returning blood from the glans. There is also a peculiar excito-motor connexion between them and the nerves of the glans, *provided* the latter be first made irritable by a partial turgescence of the vessels of the glans. If, in a dog just strangled, the glans be irritated, the bulbo-cavernosi twitch responsively, and sometimes make a rhythmical succession of contractions; and in each contraction the blood is forced from the vessels of the bulb in a rapid jet through those of the corpus spongiosum into those of the glans, which is thus each time made tense and glistening. A similar contraction of the muscles may be felt in the perineum of the dog during copulation. By the increased turgescence of the glans, the subjective sensations of its nerves is in turn increased, and thus the sensitive glans and the irritable muscular apparatus of the bulb are mutual and alternate excitors of each other. 10. The ischio-cavernosus is a hollow or bivalve-shell-shaped muscle, which covers the whole free surface of the crus penis and its bulb; its contraction, by compressing all the blood within the crus, increases and completes the turgescence of the C. cavernosum. These muscles, also, contracted with the bulbo-cavernosi as often as the turgid glans of the strangled dog was irritated; and probably in all cases the two pairs of muscles act together to increase the turgescence of the whole penis.

*Female genital organs.* In the female organs, Dr. Kobelt shows the exact analogy of the glans clitoridis to the glans penis, in more respects than are usually considered. As the analogue of the bulb he demonstrates a leech-shaped venous plexiform mass attached on each side to the ramus of the pubes, and covered by the (so-called) constrictor vaginæ muscle, the analogue of the bulbo-cavernosus; and for the analogue of the corpus cavernosum, he describes a venous plexus (*pars intermedia*) connecting the veins of each part of this bipartite female bulb with the veins of the glans clitoridis. But for the full account of these and for some more interesting matter, the book itself must be referred to.

*Uterus.* Dr. Pappenheim* has published a very minute description of the arrangement of the muscular fibres of the uterus, of which it is impossible to give a short intelligible account. For this reason alone it is that I thus pass over both it and the elaborate 'Contributions to the Physiology of the Human Ovary,' by Dr. Ritchie,† as well as the essays on the same subject by Dr. Knox.‡

*Gestation.* It is stated to be the result of researches (but no details are given) by M. Berthold,§ that after healthy gestation, delivery takes place at the time when the tenth menstruation from the time of conception should occur; in other words, when the ovary is preparing for the tenth menstruation. And hence, according as the periods of menstruation are in each case at all more or less than the ordinary time of four weeks apart, so in each case will the period of gestation be in the same proportion longer or shorter than usual. Consequently, the most probable estimate of the duration of gestation would be the number of days that elapsed during the last preceding ten menstruations.

PHYSIOLOGY OF THE OVUM AND EMBRYO.

*Separation, transit, and impregnation of the ovum.* In further evidence of the separation of the mature ova in mammalia independent of impregnation or copulation, M. Bischoff‖ has added to his observations recorded in the last Report,

* Vorläuf, Mittheib. über den Verlauf des Muskelfasern in der schwangern menschl. Gebärmutter in Roser u. Wunderlich's Archiv, and Schmidt's Jahrbucher, Mai 1844.

† Medical Gazette, Dec. 22, 1843, to June 14, 1844.

§ Report of the Académie des Sciences, Seance du 27 Mai. Arch. Gén. de Méd. Juin 1844.

‖ Beweis der von der Begattung unabhängigen periodischen Reifung und Loslösung der Eier; Giessen, 1844. Translated in the Annales des Sciences Naturelles, Août 1844; and by Mr. Henry Smith, in the Medical Gazette, Jan. 3, 17, &c. 1845.

some which are, perhaps, yet more convincing. For example, a ewe, which had never copulated, was killed twenty-four hours after the beginning of heat, and Bischoff found a Graafian vesicle ruptured in the right ovary, and the ovum in the oviduct nearly half an inch from its ovarian orifice. He cut out the left ovary of a bitch that was in heat, but had not yet copulated, and found Graafian vesicles unopened in it: five days afterwards, no copulation having been permitted, he examined the right ovary, and found four well-marked corporea lutea and the four ova far advanced in the oviduct. Similar observations were also made on a sow, and on a rat.

Dr. Ziegler* has proved that the rut of the roe is in July or August. At this time also he has found ova in the tubes; they are at the beginning of the tube towards the end of August, and at the end of it about the beginning of November. In December they pass into the uterus; on the 16th he found them as vesicles from $\frac{1}{2}$ to $1\frac{1}{2}$ lines in diameter; on the 26th the diameter was six lines. The smallest embryos were found in the beginning of January, $\frac{3}{4}$ of an inch long; in February, 2 inches; in March 6 inches long. The whole period of gestation is 40 weeks, of which three months are thus spent in the passage through the tubes. It is only from April to November that spermatozoa are found in the bucks.

Some observations by Professor Czermak† on the development of the black salamander (salamandra atra,) have interest in regard to human physiology, and especially among them these three: 1. After impregnation a certain number of ova are found in the posterior portion of each oviduct, of which one on each side is developed; but while this development is going on, a certain number of ovules grow and are developed in the ovaries; and these, after the discharge of the two young salamanders formed from the first set, pass in their turns into the oviducts, and, without a second impregnation, are developed into a second pair of young ones. Thus there is what the author calls *an incomplete super-fecundation:* a single impregnation causes two sets of ovules to be in distinct periods developed. 2. Of the ovules which pass in each period into the oviducts (or uterine horns,) although all are at first alike, yet only that one which lies on each side nearest to the cloaca is developed into an embryo; the others (*embryonotrophic ovules*), together with the albuminous envelopes which they acquire in their passage into the uterus, are fused into a pulpy, dirty-yellowish, and ropy substance, in which the elements of their yelks may be discerned. And it is on this substance that the embryo, after consuming the yelk of its own vesicle, is supported. So long as the embryo is retained in its own membranes its intestine (a simple straight pouch) is pretty full of its own yelk; but after a time it makes its way out of its membranes, and its stomach is now found distended by the yelk-substance which it has swallowed, while the greater part of the intestinal canal is empty. 3. By the time that nearly the whole of the yelk-substance in which the embryo was immersed has been consumed by it, the branchiæ have obtained the highest stage of development; and the author believes that they now serve for the absorption of a nutritive fluid, secreted from the closely adjacent vessels of the uterus, which are developed into a long-meshed network.

*Development in general.* MM. Prevost and Lebert‡ have published sundry observations on the development of the ovum of the frog, with the view, chiefly, of illustrating the development of the organs of the circulation in the batrachians and birds. In their account of the contents of the ovum before and soon after impregnation they accord with Reichert, but their account is more circumstantial. They describe the ciliated surface of the young tadpole, and the ciliæ as not set on any peculiar epithelium-cells; the pigment-cells, formed from primary-cells,

* Beobachtungen über die Bremst und den Embryo der Rehe, Hannov. 1843. Abstract in Müller's Archiv. 1844; Jahresbericht by Bischoff, who generally confirms the account.

† Oesterreichische Medicin. Jahrbucher, Oct. 1843.

‡ Annales des Sciences Nat. Avril 1845. Possibly there is more of novelty in this paper than I have stated; but the account given is obscure, and is made the more so by the adoption of new names, and the avoidance of any reference to those by which others have called the same things. In a following number of the Annales is a paper of the same kind, on the development of the chick. See also the remarks by Vogt on the former paper in the same Journal. July, 1844.

(the "smaller predisposed vitelline-cells" of Reichert, the "organo-plastic globules" of Prevost and Lebert,) of which some, in the pigmentum nigrum, retain their form, and others become star-like, and form canal-communications; the development of muscular fibres from similar cells elongating themselves in groups, and having their granules developed into muscular filaments; and the development of the chorda dorsalis. In regard to the development of the blood-corpuscles they confirm the account of Baumgärtner, Schultz, Reichert, and Bischoff, that in the frog, they are formed, like the tissues, out of the smaller vitelline-cells, which gradually pass from the round to the oval form, have their contained granules resolved, and acquire the red colouring matter. The development of the heart is described as by Reichert and others; and in the first formation of blood-vessels, they believe that the cells and other constituents of the tissue in which they are to be formed separate so as to leave the spaces which are to be occupied by the vessels. The capillaries, they add, are never formed independently of the general circulation, but always centrifugally under the influence of the circulation, by arches passing from a minute artery to a corresponding vein.

*Development of the tissues. Of bone.* Bidder* has given a minute account of the development of bone, from examinations of the long bones of kittens just born. In vertical sections, he finds just below the synovial membrane on the head of the bone, six or eight layers of the usual flattened cartilage-corpuscles; below these is a layer of cells of irregular shape and size, irregularly and thinly scattered in the intercellular substance, with clear, dark outlines, and finely granular contours; nearer the ossified part the corpuscles are almost all oblong, larger, proportionally more numerous, and they have more distinct granules, which in some have assumed the form of distinct nuclei. After these, in the layer immediately adjacent to the bone, the corpuscles, as Mieschaer showed, are arranged longitudinally in rows vertical to the ossified surface; they are oblong, with their long axes transverse to the direction of the rows, and in the several rows are very closely set. The smallest of them retain their finely-granular or grumous contents, and appear opaque and jagged, or crenate at their margins; others, and these always the larger, are clear and contain bright nuclei. The last layer of cartilage next the bone is further distinguished. The rows of corpuscles are less regular. (The nuclei and the contents of the cells are so alterable that they must be examined directly after death, especially in young animals.) The corpuscles, as in the previous row, may be divided into two kinds, and they occupy three-fourths of the whole substance. Some are large cells with one, two, or three clear distinct nuclei, and are either oblong or tolerably round or angular, with, sometimes, a double contour. Their nuclei also contain from one to four nucleus-corpuscles. The cells of this kind are formed by the gradual fusion of two, four, or more of the clear corpuscles last described, which when they first coalesce, form dentated dark corpuscles with granular contents, but gradually clear up, become rounder, obtain the distinct double contour, and the one or more nuclei; that is, from the conflux of several cartilage cavities, there are formed complete cells with nuclei and nucleoli. Others of the corpuscles at this part have opaque granular contents and irregularly crenated edges, and agree pretty nearly with the crenate-cells before mentioned; and these may sometimes appear to be inclosed in cells of the same kind as the clear nucleated ones last described; but they are certainly distinct and independent.

In the ossified part it is clear that the ossification first makes progress in the intercellular substance between the vertical rows of cells, and then passes transversely between them, so as to form cancelli, each of which incloses one or more of the true enlarged and nucleated cartilage-corpuscles; while the dentated corpuscles are themselves inclosed in the deposits and impregnated with the calcareous matter. (In different specimens, however, the degrees of regularity of the arrangement of these cartilage-corpuscles are very uncertain, and many must be examined to afford the complete view.)

* Müller's Archiv, Heft iv-v, 1843.

It thus appears that of the original cartilage-corpuscles, some are developed and some retrograde. The former increase to three times their original size, at first by growth, and at last by coalescence; their walls, at first not distinguishable fro[illegible] the intercellular substance, become thicker and more distinct, crenated, and then round with a double contour; their contents, at first clear, become turbid and granular, and then again clear, with distinct nuclei and nucleoli; they arrange themselves in regular rows, and seem to grow at the expense of the intercellular substance.

The latter (retrograding) corpuscles are those which do not attain a higher grade than that of the dentated and crenated corpuscles; they are smaller than the former, their dark granular contents give no indication of the formation of new cells, they do not grow, and at last are ossified together with the intercellular substance.

The intercellular substance does not in these changes remain unaltered. Before ossification, it becomes laminated in the large-celled layer of cartilage immediately adjacent to the ossified part; and this laminar arrangement is the forerunner of the osseous lamellæ, which in a later stage surround the vascular (Haversian) canals. These vascular canals are unquestionably produced by the fusion of the cancelli formed from the more perfect cells: they are not derived from canals previously formed in the cartilage, but result from the absorption of the partitions between cancelli, of which some lay in longitudinal, others in oblique or transverse rows. The canals in cartilage, from which those of bone were supposed to be formed, are far from constant in ossifying cartilage, even when ossification has made much progress, and when they do exist they scarcely ever reach so far as the ossifying part, but stop short near its surface.

The other kind of corpuscles, the small dentated ones, become the bone-corpuscles as earthy matter is deposited all around them; and the author inclines to Henle's view, that the *canals of the corpuscles* are formed as *intercellular spaces* in substance deposited in laminæ within the corpuscles. But in a note appended to the paper, Müller says that in enchondroma he has often seen the transition from the nucleus of the distinct cell into the toothed and branched nucleus. So that, as Bidder himself admits, this part of the subject must be still very doubtful.

*Development of arteries.* H. Rathke* has published a very complete account of the development of the arteries which proceed from the arch of the aorta in mammalia and man. His examinations were made on embryos of pigs, sheep, and oxen.

When the pharyngeal (or branchial) fissures are still open, the heart lies behind and below them; its single ventricle is continued anteriorly into a single canal, which corresponds with the trunk of the branchial arteries of fish, but has no bulb; this, as it passes forwards, gives off or divides into five arterial arches on each side, of which four pass between the pharyngeal fissures, and the fifth passes behind the last fissure. Above the fissures all these arches unite into one aorta, which thus, as in fish, arises with two divided roots from each side, and is continued down the vertebral column.

When the ventricular septum has formed, the single trunk proceeding from the originally single ventricle also divides into two trunks, partitions continuous with that of the ventricle growing from opposite parts of its walls and uniting within it. Of these two trunks, the right passes at its further end rather behind the left; and when the partition between them is completed, the left trunk appears as the ascending aorta, the right as the trunk of the pulmonary artery, which, enlarging at its lower (or proximal) part forms the *conus arteriosus,* and here constitutes a part of the heart itself. No similar enlargement of the aorta ever takes place.

But before these changes are finished, it is found that of the five arterial arches on each side, the anterior pair (the most distant from the heart) disappears almost entirely: but the proximal portion of each of these arches, from which there is already given off a branch to the brain, remains; and the remnant of the trunk, together with this branch, now appears like a branch from the second arch. A little later the second pair of arches also disappears, their proximal portion only

* Müller's Archiv, Heft iii-iv, 1843.

remaining ; and the same is soon repeated with the third pair of arches. Thus, there now remain only the fourth and fifth pairs of arches, and a vessel formed on each side out of parts of the first three pairs, which looks like a branch given off from the enlarged and now more divergent fourth pair. This vessel is the common carotid ; it is S-shaped, having a proximal curve turned towards the mouth, and a distal curve directed backwards; a branch is given off from it near its origin, and passes to the anterior fissure, where the ear is formed. This branch is the facial (external) carotid; the continued trunk, including the branch before mentioned as given off from the first arch, is the cerebral (or internal) carotid.

All the fissures, except the anterior, are now closed; the partition of the single arterial trunk from the heart is soon completed; and at length the fourth and fifth pairs of arches having increased greatly in size and strength, the right ventricle sends all its blood into the fifth pair, the left into the fourth pair and the carotids.

The present condition of parts, therefore, is this. 1. The trunk from the left ventricle divides into two branches, which go to each side, and ascend arching upwards and backwards, then run converging for some distance further backwards, and, between the œsophagus and vertebral column, pass at an acute angle into the trunk of the (descending) aorta. Of these branches, the right is, in its whole length, but especially towards its distal end, smaller than the left, which is to be developed into the arch of the aorta, and its convex portion lies a little further forwards than that of the left does. Each of these branches, also, at some distance from its origin, gives off a carotid branch. 2. The current from the right ventricle, passing into the trunk of the pulmonary artery, in like manner divides into two branches, which ascend arching upwards and backwards, and of which the right is smaller and rather more anterior than the left. Both these branches are placed behind those of the former trunk, into which, at their distal ends, they pass at an acute angle, and than which they are rather smaller.

The fifth arch, on the left side, now gives off a branch which goes backwards to the lungs, and ramifies in them. This branch becomes the distal half of the trunk of the pulmonary artery ; the proximal part of the same left fifth arch becomes the proximal half of the pulmonary artery; and the distal part of the arch (that part which is beyond the branch) becomes the ductus arteriosus. The fifth arch of the right side gives off no such branch, and gradually wastes away.

The vertebral arteries arise very early from the distal part of each fourth arch just before it passes into the dorsal aorta : that of the left side arises just before the junction of the ductus arteriosus with the fourth arch. They run up on the six upper vertebræ, and soon became inclosed in short double processes (the cervical transverse processes) which unite, first together and then with the vertebræ, and form foramina. On the left side the vertebral artery sends off a branch which goes far backwards to the anterior extremity, and constitutes the distal half of the left subclavian artery, of which the proximal half is afterwards formed by the lower part of the vertebral enlarging so much, in correspondence with the development of the anterior extremity, that its upper half appears like a branch from it. The right subclavian is given off as a branch from the distal half of the right fourth arch, at some distance beyond the origin of the right vertebral from the same. It also bends far backwards to reach the right anterior extremity.

The part of the fourth arch on the right side, which lay beyond the subclavian branch just mentioned, next wastes away, and the rest of this arch, having grown less than the corresponding arch on the left side, with which it is connected at their common origin from the aortic trunk, now assumes the appearance of an arteria innominata given off from the commencement of the left fourth arch. This left fourth arch, together with the aortic trunk, thus becomes the proper arch of the aortic, giving off, first, the innominata, then the left carotid, and then the branch from which the left vertebral and subclavian are derived.

After this, probably, the only important alterations which take place in the human embryo, are the change in the direction of the subclavian arteries as the heart retreats further into the cavity of the chest, the elongation of the carotids in correspondence with the growth of the neck, the enlargement of the common trunk of the left subclavian and left vertebral, so that the former, which at first appeared like a branch of the latter, now appears like a trunk giving it off, and the elongation of that part of the arch of the aorta which lies just before the origin of the left subclavian.

In the embryos of the mammalia which were examined, further changes effect the coalition of the three branches from the arch of the aorta into two or one; and the plan of these changes is pointed out at the end of the essay.

*Development of the nervous system.* From Bischoff* we have some further observations on the development of the nervous centres, which confirm his own and V. Baer's former descriptions, and add to them some interesting minute details. In the examination of four ova, from a bitch which had conceived three weeks previously, he found, in the first, the dorsal laminæ formed by accumulations of formative substance on each margin of a groove (the *primitive groove* or *streak*) which lay in the long axis of the pyriform germinal area, and was not covered in by even the most delicate membrane. In the second and third ova which were removed from the living uterus twenty-four hours after the removal of the first, the groove was still open, although the rudiments of six vertebral arches were formed in the dorsal laminæ on each side of it; at the head-end its margins were separated in three successive dilatations, in the middle they were nearly in contact, and at the tail-end they had a lancet-form; and, which was most interesting, the inner borders of these dorsal plates, and thus the inner layer of the substance forming the primitive groove, presented distinctly the transparent aspect of nervous matter. In a fourth ovum, removed from the uterus twelve hours later, the groove was almost all closed in, and the borders of the nervous matter having united along almost their whole length, presented the appearance of a medullary tube, dilated and bent downwards anteriorly, and having the sacculi for the eyes considerably developed.

The conclusion, therefore, is that of the substance which accumulates by the sides of the primitive groove, and forms the dorsal laminæ, the inmost layer forms the nervous tube which is the rudiment of the central nervous system; that the rest of this substance is developed into the dorsal part of the trunk; and that the nervous substance is formed even before the closing in of the groove.

*Respiration of the embryo.* The researches of MM. Baudrimont and Martin St. Ange† on the chemical changes produced in the incubation of the eggs of birds have shown that in all cases oxygen is absorbed and water and carbonic acid are given off by the eggs. The evolution of these substances appears essential to the progress of development; and they are probably derived from the combustion of carbon and hydrogen within the ovum. The quantity of carbonic acid which is given off increases with the period of impregnation; that of water is nearly the same at all the periods. From this combustion moreover eggs must have a proper temperature independent of that communicated by the mother, and the authors suggest that it might have been in part the source of the increased heat (amounting to from 12° to 19° above the surrounding temperature,) observed by M. Valenciennes‡ to be developed during the incubation of the eggs of the boa (python birittatus.)

*Food of the embryo.* Experiments by Signor Capezzuoli§ appear to prove that the fatty matter of the vitellus is especially consumed by the chick during the last days of incubation; and that much of that contained in the chick itself is consumed by it shortly after it is hatched.

*Excretions of the embryo.* Dr. John Davy|| has analysed the meconium and vernix caseosa. The former contains

| | |
|---|---|
| Mucus and epithelium scales . . . | 23·6 |
| Cholestearine (in plates,) and margarine . . | ·7 |
| Colouring and sapid matter of bile, and oleine . . | 3 |
| Water . . . . . | 72·7 |

* Müller's Archiv, 1843, Heft iii-iv.

† Annales des Sciences Naturelles, t. xvi, p. 65.

‡ Report from the Académie des Sciences, Seance de Dec. 26, in the Gazette Médicale, Dec. 30, 1843, and confirmed by more recent and numerous experiments, in the Report from the Academy for December 16, 1844.

§ Annali univ. di Medicina; Gennaio, 1844; see also the foregoing observations by Czermak.

|| Medico-Chirurgical Transactions, vol. xxvii, p. 189. 1844.

Its ashes also contained peroxide of iron and magnesia, with a trace of phosphate of lime and chloride of sodium.

In the vernix caseosa he has found,—

| | |
|---|---|
| Epithelium (epidermis,) plates | 13·25 |
| Oleine | 5·75 |
| Margarine | 3·13 |
| Water | 77 37 |

and in the very minute quantity of ashes which remained he found traces of phosphate of lime and sulphur.

---

The subjoined list includes references to the more valuable of the physiological Essays published last year, of which, for various reasons, no notice is contained in the body of the Report. They are arranged according to the subjects to which they relate, in the same manner as the Report itself.

Prof. Harting—Observations on the original forms and subsequent alterations of organic and inorganic precipitates; on the phenomena of the formation of crystals; &c. in the Tijdschrift voor Naturel. Geschied. en Physiologie, 1843, St. 2, 3.

Mr. T. W. Jones—Muscle a neuro-magnetic apparatus, in the Medical Gazette, Oct. 20, 1843; and Dr. Letheby's Remarks on the same in the Physiological Journal, Dec. 1843.

Dr. Houston—On the circulation in acardiac fœtuses; in the Dublin Journal of Med. Sciences, Jan. 1844; and Dr. Marshall Hall's reply, in the same, Sept. 1844.

Dr. Thompson, Prof. Draper, and others—On the diffusion of gases, in the Philosophical Magazine, July 4, 1844.

Mr. Sibson—On the changes induced in the situation and structure of the internal organs in health and disease; in the Trans. of the Prov. Med. and Surg. Association, vol. xii, 1844; [a very important paper, the title of which led me to suppose, till it was too late to correct my error, that it could have little interest in physiology.]

S. Rusconi—On the respiratory organs of the proteus. Oken's Isis, viii, 1844.

Prof. Nasse—On the application of percussion to the study of gastric digestion; in the Med. Corresp.—Blatt. Rhein. und Westphal. Aerzte. No. xvii, 1843.

Dr. Heintz—On a supposed new acid contributing to give the acid reaction of urine; in Poggendorf's Annalen, No. viii. 1844.

M. Parise—On a new method of bleaching bones, by alkaline and alcoholic injections of their texture through small holes bored in their walls; in the Ann. de la Chirurgie, Fevr. 1844.

Dr. Todd—The article *Nervous Centres,* in the Cyclop. of Anatomy.

M. Flourens—The new edition of his researches on the nervous centres.

Dr. Wigan—On the duality of the mind; in the Lancet, March 30, 1844.

Dr. Playfair—On sleep and some of its concomitant phenomena; in the Northern Journal of Medicine, May, 1844.

M. de Haldat—The summary of his researches on the crystalline lens; and M. Prevost, on single vision with two eyes; in Poggendorf's Annalen, 1844, Heft viii.

S. Rusconi—On the non-erectile tissue of the tongue of the chamelion and its protrusion by a muscular apparatus; in the Ann. des Sciences Naturelles. Mars 1844.

Dr. J. Y. Sympson—On the fruitfulness of females born co-twins with males; in the Edinburgh Med. and Surg. Journ. Jan. 1844.

M. Serres—On the corpora Wolffiana and allantois [referred to in the last Report,] in the Ann. des Sciences Nat. 1843. p. 4, Pt. i.

Lund—On fossilized human bones in chalk caves in Brazil, [the caves contain bones of several extinct species, but admit each year the waters of adjacent lakes;] in the Edinb. New Philos. Journal. Jan. 1844.

Various papers on anthropology and ethnology, from the proceedings of the Ethnological Society; in the numbers of the Edinb. New Philos. Journal.

Dr. Laycock—Contributions to proleptics, in several numbers of the Lancet. A summary of them was given in this Journal, July 1844.

